# Salivary Microvesicle Methylome and Microbiome Profiles in Periodontitis: An Exploratory Study

**DOI:** 10.1111/jcpe.70114

**Published:** 2026-03-26

**Authors:** Pingping Han, Chaminda Seneviratne, Qiongyi Zhao, Carlos Salomon, Xiang Li, Sašo Ivanovski

**Affiliations:** ^1^ School of Dentistry, The University of Queensland Brisbane Queensland Australia; ^2^ Center for Orofacial Regeneration, Rehabilitation and Reconstruction (COR3), Epigenetics Nanodiagnostic and Therapeutic Group, The University of Queensland Brisbane Queensland Australia; ^3^ The University of Queensland, Queensland Brain Institute Brisbane Queensland Australia; ^4^ Faculty of Medicine, Translational Extracellular Vesicles in Obstetrics and Gynae‐Oncology Group, Centre for Clinical Diagnostics University of Queensland Centre for Clinical Research, Royal Brisbane and Women's Hospital, The University of Queensland Brisbane Queensland Australia; ^5^ Department of Anesthesiology, Brain Research Center, Department of Neurosurgery, Zhongnan Hospital Wuhan University Wuhan Hubei Province China

## Abstract

**Aim:**

Salivary microvesicles (MVs) are nanosized extracellular vesicles from the host and microbiota whose cargo may mirror the biological state of their parent cells. This cross‐sectional study aimed to explore the diagnostic power of host methylome and microbiome profiles of MVs in various periodontal disease states.

**Materials and Methods:**

This exploratory study recruited 20 healthy, 16 gingivitis and 26 stage III/IV periodontitis cases. The origins of salivary host‐MVs were identified using a multiplex extracellular vesicle (EV) kit. The microbiome and host methylome profiles of MV DNA were analysed using 16S rRNA sequencing and methylated DNA immunoprecipitation sequencing (MeDIP‐seq), respectively.

**Results:**

The periodontitis group showed increased CD63+, CD45+, CD29^+^ and CD24^+^ MV subpopulations (AUC > 0.7), along with significantly higher bacterial outer membrane vesicles (AUC > 0.89) from *Treponema*, *Fretibacterium* and *Treponema denticola* , compared to both healthy and gingivitis groups, as well as the non‐periodontitis (combining healthy and gingivitis) group. MeDIP‐seq identified 1196 differentially methylated regions across 3′ UTRs, CDS, introns and intergenic regions (AUC > 0.9), distinguishing periodontitis from the other groups. These methylated genes were enriched in inflammation‐related pathways, including AMP‐activated protein kinase (AMPK) and Toll‐like receptor 4 (TLR4) pathways.

**Conclusion:**

This exploratory study found that host methylome and microbiome profiles in salivary MVs reflect periodontal disease status, and hence supports their potential as non‐invasive liquid biopsy biomarkers for periodontitis.

## 1 Introduction

Periodontitis, a chronic inflammatory oral disease, is caused by a microbial dysbiosis associated destructive host immune response, where the interplay between dental plaque, genetics and epigenetics leads to irreversible loss of tooth‐supporting tissues (Hajishengallis and Chavakis 2021; Lamont et al. 2018; Loos and Van Dyke 2020). Current clinical diagnosis relies on detecting past tissue damage (i.e., probing pocket depth [PPD], clinical attachment loss [CAL]) (Salvi et al. 2023), with no reliable indicators of real‐time disease activity. Precision periodontics using integrated omics has been proposed to identify biomarkers for periodontitis (Bartold and Ivanovski 2022), with the latest disease classification framework recognising the potential of biomarker‐driven diagnostics (Tonetti et al. 2018). Although various salivary cytokines and proteins have been suggested as biomarkers (Blanco‐Pintos et al. 2023), the methylome and microbiome landscapes of salivary extracellular vesicles (EVs) remain largely unexplored despite their promise in reflecting microbe–host interactions and contributing to precision periodontics as alternative diagnostic tools (Bartold and Ivanovski 2022).

Saliva is a promising non‐invasive diagnostic fluid for periodontitis (Giannobile 2012; Han et al. 2023; Han et al. 2022), as it contains local and systemic biomarkers of both host and microbial origins, such as proteins, genetic material, EVs and microbial by‐products. Among these, epigenetic markers found in both whole saliva and salivary extracellular vescicles (EVs)—such as DNA methylation, histone modifications and non‐coding RNAs—are emerging as key contributors to periodontal disease pathogenesis (Liaw et al. 2023; Liaw et al. 2022; Liaw et al. 2024; Liu et al. 2025; Liu et al. 2023; Suzuki and Yamada 2022; Han et al. 2025). EVs are lipid bilayer–enclosed particles present in biofluids, such as saliva, carrying bioactive molecules of host and microbial origins (Han et al. 2022; Liu et al. 2024). Based on their biogenesis, EVs are typically classified into three subtypes: apoptotic bodies, microvesicles (MVs) and small EVs (sEVs, or exosomes, derived from the endocytic pathway), which overlap in size (Buzas 2023). Studies have shown that salivary exosomes (< 200 nm) can serve as a liquid biopsy biomarker for periodontal status, offering greater accuracy in reflecting periodontal disease status than whole saliva (reviewed in (Han et al. 2022; Cui et al. 2024)). However, MVs (50–1000 nm), which are produced by direct membrane budding and contain more abundant genomic DNAs than small cell–free DNA in exosomes (Balaj et al. 2011; Vagner et al. 2018), particularly those recovered after the 16,000*g* centrifugation step, remain less studied and warrant further investigation.

**FIGURE 1:**
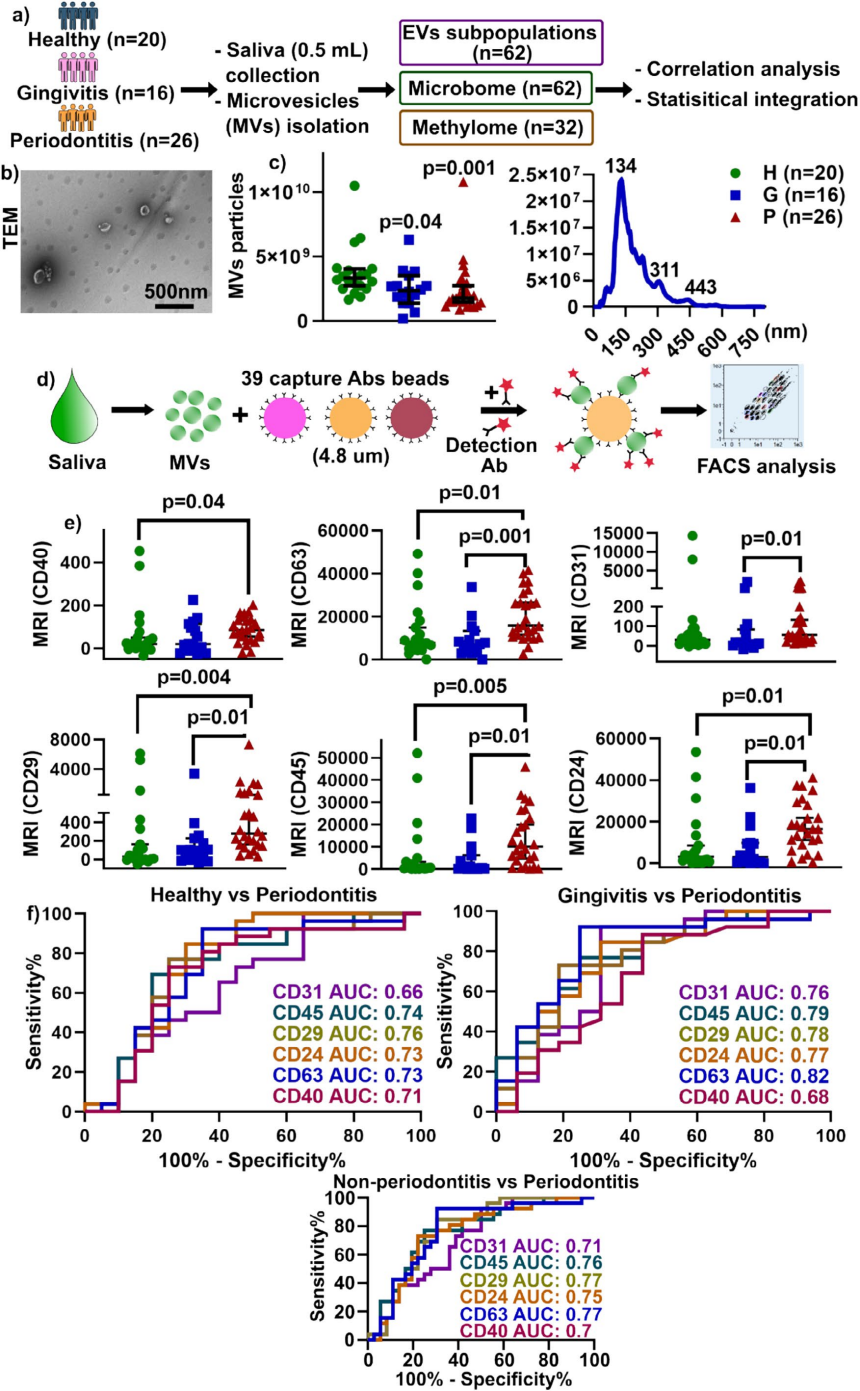
Summary of study design and EV subpopulations of salivary MVs distinguishing periodontitis from healthy participants. (a) Samples were collected from 20 healthy, 16 gingivitis and 26 periodontitis participants and type of omics and downstream analysis. (b) A representative TEM image of isolated salivary MVs. (c) Characterisation of MVs using nanoparticle tracking analysis with MV particle numbers (left) and representative histogram for gingivitis‐derived MVs. (d) Workflow of a multiplex bead‐based assay for the detection of EV surface markers. Thirty‐seven multiplexed populations of dye‐labelled antibody‐coated capture microbeads were incubated with EV samples. Ab: antibody. (e) Representative quantification of background‐corrected APC median signal intensities (MRI) for CD 40, 63, 31, 29, 45 and 24 bead populations. Data are displayed as scatter dot plots with a median + 95% CI. (f) ROC curve analysis of various CD markers in periodontitis patients compared to healthy or gingivitis, or non‐periodontitis (combining healthy and gingivitis) patients. *p*‐values are from the Kruskal–Wallis test with Dunn's multiple comparisons test (c‐left, e). Graphs in (c) and (e) are presented as scatter plots with median ± 95% CI.

A few studies have employed a multi‐omics (i.e., methylome and transcriptome) approach to identify clinical biomarkers in plasma and urine (Schüssler‐Fiorenza Rose et al. 2019; Tebani et al. 2020; Zhou et al. 2019); for example, increased salivary tumoural MV particles and CD8+/EGFR+ salivary MVs have been reported as diagnostic and prognostic indicators for oral squamous cell carcinoma (Zhong et al. 2019; Man et al. 2023). To date, no studies have examined salivary MVs as biomarkers integrating both microbiome and host methylome profiles, which is advantageous for capturing both aspects of periodontitis pathology. This exploratory study is the first to characterise both microbiome and host DNA methylome profiles of salivary MVs in a clinical cohort (*n* = 62) of healthy (H), gingivitis (G) and periodontitis (P) patients (Figure 1a), with the aim of evaluating their potential as novel biomarkers and diagnostic tools for precision periodontics.

## 2 Materials and Methods

### 2.1 Participant Recruitment

All participants in this study provided informed written consent, and the research received ethical approval from both Metro North Hospital and Health Service and the University of Queensland (Ethics App. No. 54584 and 2018001225). The study adhered to the principles outlined in the Declaration of Helsinki concerning experimentation involving human subjects. A total of 116 patients were recruited for patient screening (Figure S1 and detailed in Supporting Information).

### 2.2 Salivary MV Isolation and Characterisation

Unstimulated whole saliva was collected from participants before their routine appointments (between 9 AM and 12 noon), after at least 1 h of avoiding drinking, eating, using mouthwash or chewing gum, following our previously published protocols (Liu et al. 2023).

Salivary MVs were isolated using serial centrifugation as detailed in Supporting Information. MVs underwent characterisation following the guidelines from the Minimal Information for Studies of Extracellular Vesicles 2023 (MISEV2023) (Welsh et al. 2024), which included assessing their morphology using transmission electron microscopy (TEM), identifying origins of host MVs using the human MACSPlex Exosome Kit, determining particle number and size through nanoparticle tracking analysis (NTA) and employing Fourier transform infrared spectroscopy (FTIR).

### 2.3 Origin of Host MV Sub‐Populations Using a Multiplex Platform

The analysis of 37 surface markers of EVs and distinct subpopulations of MVs was conducted with a human MACSPlex Exosome Kit (Miltenyi Biotec, Bergisch‐Gladbach, Germany) as per the manufacturer's instructions.

### 2.4 Salivary MV DNA Isolation

MV DNA isolation is detailed in Supporting Information. The quality of DNA was assessed by the Qubit dsDNA HS assay (Invitrogen) and the DNA high‐sensitivity chip on an Agilent 2100 bioanalyser (Agilent Technologies).

### 2.5 MV Microbiome by 16S rRNA Sequencing

The microbiota‐derived bacterial EVs were employed in DNA sequencing targeting the V3 and V4 hypervariable regions of the 16S rRNA gene. This sequencing was conducted using an Illumina MiSeq instrument (Illumina, San Diego, CA, USA) in conjunction with proprietary primers designed by GENEWIZ. Differentially abundant microbial biomarkers at the genus level were identified using Metastats, with a false discovery rate (FDR) threshold set at < 0.05. Detailed statistical analyses and visualisation of diversity metrics were performed using R.

### 2.6 Sensitive Host DNA Methylome Profiling Using MeDIP‐Seq

We analysed global DNA methylation, including 5‐methylcytosine (5mC), 5‐hydroxymethylcytosine (5hmC) and N6‐methyladenosine (m6dA), using DNA samples obtained from 20 healthy, 16 gingivitis and 26 periodontitis individuals, as per the manufacturer's instructions from Abcam.

Figure S6e illustrates a schematic depiction of the MeDIP NGS‐seq protocol. The global epigenome profile was then compared from selected patients of 9 healthy, 12 gingivitis and 11 periodontitis groups using edgeR (v 3.28.1) with FDR < 0.05. A commonly methylated gene network was performed using the GeneMANIA software (v3.6.0). Enrichment of differentially methylated regions (DMRs) of genes was analysed for function clusters, Gene Ontology and pathways using DAVID (v6.8) (Huang et al. 2009).

### 2.7 Statistical Analysis

The data are presented as a median ± 95% confidence interval (CI). Normality was tested with the D'Agostino–Pearson method, and non‐parametric differences were analysed using Kruskal–Wallis test with Dunn's multiple comparisons (GraphPad Prism 10.1). Receiver operating characteristic (ROC) curves and area under the curve (AUC) were generated to demonstrate the diagnostic power of each investigated parameter in GraphPad Prism 10.1. Detailed analysis is given in the Supporting Information.

## 3 Results

### 3.1 Study Cohort and Salivary MV Characterisation

The clinical periodontal health status of participants is shown in Table 1. In the present study, 62 age‐matched participants were recruited (Figure 1a), consisting of 20 healthy (denoted as H), 16 gingivitis (denoted as G) and 26 stage III/IV generalised periodontitis (denoted as P) (Figure S1) individuals. MV characterisation was performed using TEM (Figure 1b) and NTA (Figure 1c). Representative size distribution of MVs in the H, P (Figure S2c) and G groups showed several peaks, with the highest peak at ~130–150 nm.

**TABLE 1:** Demographic data of the 62 clinical patients for this study.

	Healthy (*n* = 20)	Gingivitis (*n* = 16)	Periodontitis (*n* = 26)
Gender			
Male	7 (35%)	9 (53%)	12 (46.15%)
Female	13 (65%)	7 (41%)	14 (53.85%)
Age (years)	56.5 ± 16.1 (24–78)	45.6 ± 21.4 (22–89) (*p* = 0.106)	52 ± 9.8 (36–69) (*p* = 0.8047)
Ethnicity			
Caucasians	91%	62.5%	69%
Asians	9%	37.5%	31%
BOP (%)	7.39 ± 2.57	44.2 ± 18.3 (*p* < 0.0001)	56.03 ± 21.37 (*p* < 0.0001)
PI (%)	13.14 ± 9.86	26.31 ± 32 (*p* = 0.0313)	26.67 ± 31.92 (*p* = 0.0017)
No. of deep pockets (≥ 5 mm)	0	0	32.78 ± 18.2
PISA (mm^2^)	67.8 ± 40.04	478.9 ± 97.7	1270 ± 529.9 (*p* = 0.0001)
Average PPD (mm)	1.98 ± 0.26	2.4 ± 0.32	4.92 ± 0.88 (*p* < 0.0001)

*Note: p*‐values were calculated versus healthy group using an unpaired Mann–Whitney *t*‐test; data are displayed as mean ± SD.

### 3.2 Salivary Host MV Sub‐Populations Using a Multiplex Microbead Platform

All 37 EV surface markers were identified (Figure 1d) and are displayed in Figure S3a. The common EV tetraspanin markers—CD9, CD81 and CD63—were not evenly distributed among salivary MVs, with CD63 being more abundant than CD9 and CD81 (Figure S3a).

The P group also had significantly higher expression of CD40+, CD63+, CD31+, CD29+, CD45+ and CD24+ salivary MV sub‐populations compared to the G group (Figure 1e). These six proteins were shown as a network in Figure S3b, indicating that the MVs in the P group likely originate from antigen‐presenting cells (CD40+), platelets/lymphocytes (CD29+), endothelial cells (CD31+), haematopoietic cells (CD45+) and epithelial cells (CD24+) during the pathogenesis of periodontitis.

The analysis of ROC curves and AUC for the six CDs proteins showed lower AUC values, less than 0.6 between the H and G groups (Figure S3c). On the contrary, there was significantly higher AUC (> 0.7) in the P group in comparison to the H and G and non‐periodontitis (H + G) groups (Figure 1f). It is noteworthy that CD63+ MVs had a significantly higher AUC of 0.82 between the P and G groups.

### 3.3 Detection of Bacterial EV in Saliva for Periodontitis

**FIGURE 2:**
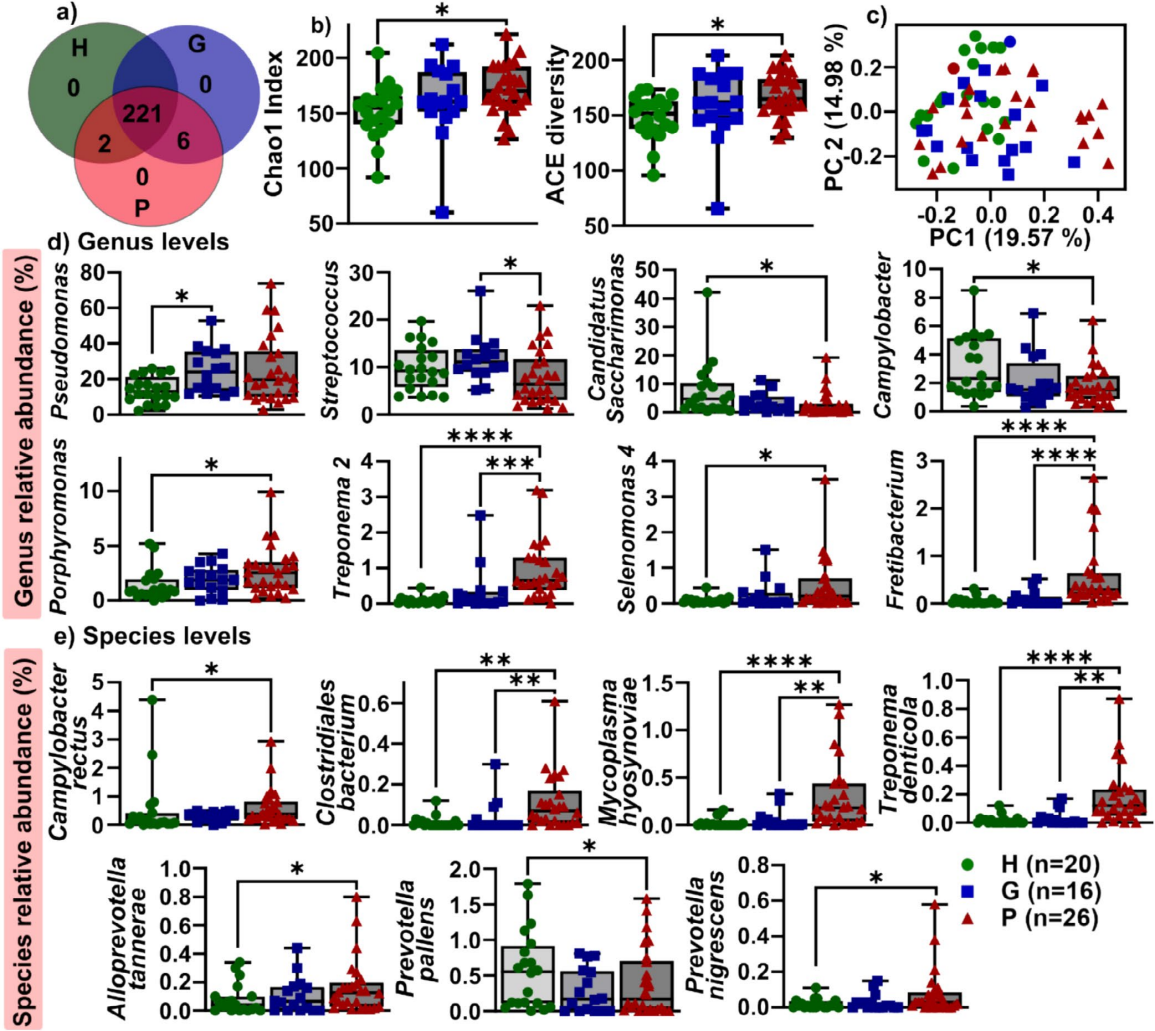
The microbiome profiles of salivary MVs in periodontitis. (a) Venn diagram of OTUs in healthy‐, gingivitis‐ and periodontitis‐derived salivary MVs. (b) The α diversity of Chao 1 and ACE changes in periodontitis. *p*‐values are versus healthy using the Kruskal–Wallis test with Dunn's multiple comparisons test. (c) Principal co‐ordinates analysis (PCoA) of OTU‐based data analysis using the Bray–Curtis distance matrix method. (d, e) Significant genus (d) and species (e) expression across the three groups. Data are displayed as box and whiskers graphs in (b), (d) and (e). *p < 0.03, **p < 0.002, ***p < 0.0002, ****p < 0.0001 between groups in (b) and (d–f), as determined by the non‐parametric Kruskal–Wallis test with Dunn's multiple comparisons. Data normality was assessed using the Anderson–Darling test, and all datasets showed a non‐parametric distribution.

After the V3–V4 region of 16S rRNA sequencing, six OTUs were observed only in the G and P groups: *Candidatus Saccharimonas*, *Acholeplasma*, *Moraxella*, *Treponema*, *Roseburia* and *Proteobacteria*, whereas two OTUs (*Rikenellaceae* and *Campylobacter*) were specific to the H and P groups (Figure 2a). The heatmap showing the distribution of the top 30 OTUs and their relative abundance is shown in Figure S4d–f. No difference in the Shannon index, but significantly higher Chao 1 and ACE alpha diversity, was detected in P compared to H samples (Figure 2b and Figure S5a). There was no significant beta diversity between the three groups in terms of principal coordinate analysis (PCoA; Figure 2c and Figure S5b).

**FIGURE 3:**
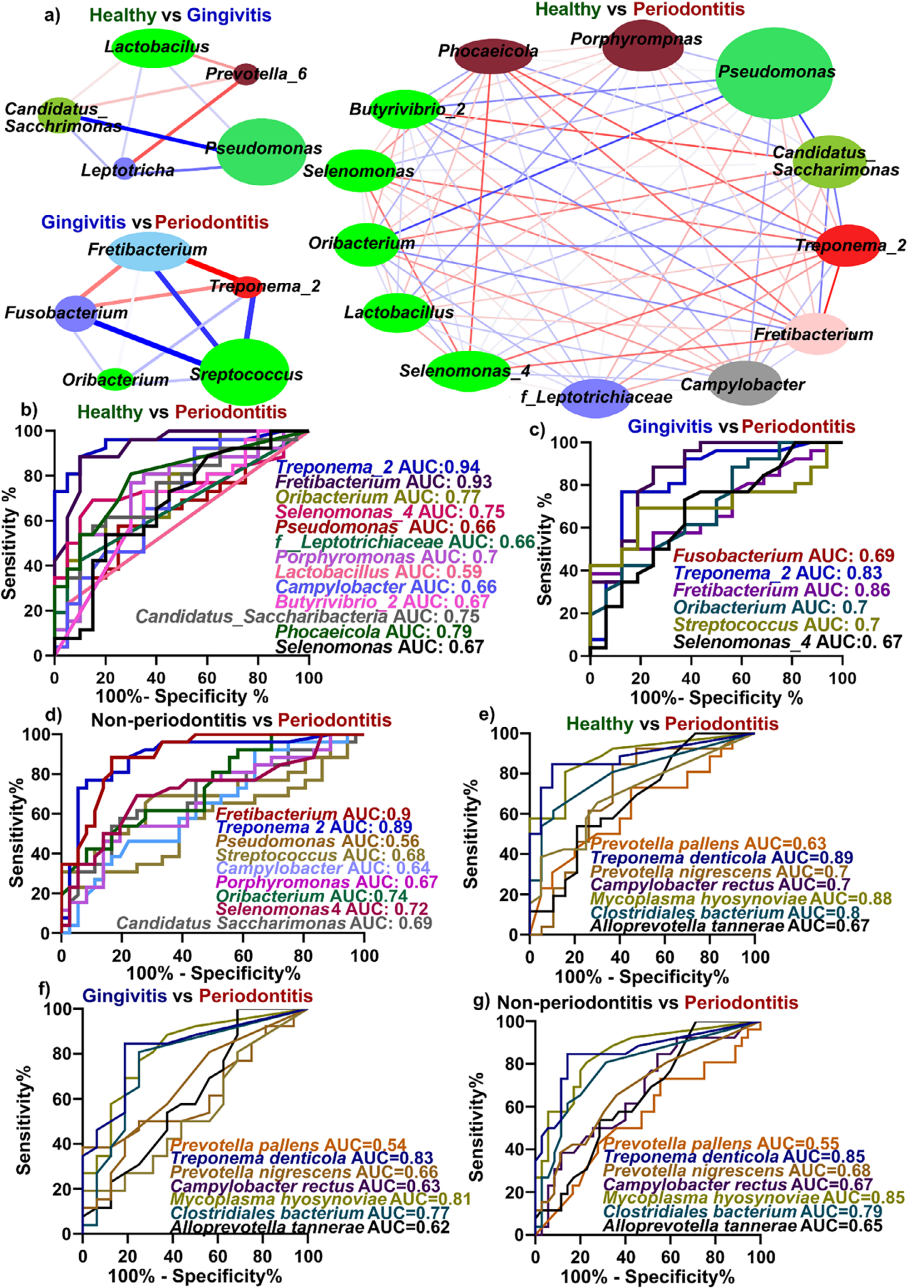
Microbiome network analysis and discrimination power of differentially expressed genera. (a) Network analysis of microbial genus community structure and function for different diseases. Cytoscape 3.5.1 was employed for the analysis, wherein distinct nodes represented various genera, and the size of each node reflected the average relative abundance. The thickness of the connecting lines between nodes correlates positively with the absolute value of the correlation coefficient of species interaction. A red line signifies a positive correlation, while a blue line indicates a negative correlation. (b–d) ROC curves showing the diagnostic power of differentially expressed genus to distinguish periodontitis from both healthy (b), gingivitis (c) and non‐periodontitis (combining healthy and gingivitis) (d). (e–g) ROC curves of differentially expressed species between periodontitis and healthy (e), gingivitis (f) and non‐periodontitis (combining healthy and gingivitis) (g).

Bacterial EVs from the genus *Pseudomonas* was enriched in gingivitis, whereas BEVs from *Candidatus Saccharibacteria*, *Campylobacter*, *Porphyromonas*, *Treponema 2* and *Selenomonas 4* were significantly enriched, and *Fretibacterium* was decreased in periodontitis compared to healthy controls (Figure 2d). Increased BEVs from *Treponema 2* and *Fretibacterium* and decreased *Streptococcus* were found in the P group compared to the G group (Figure 2d). In terms of species, in P compared to H controls, BEVs from *Campylobacter rectus* , *Mycoplasma hyosynoviae* , *Alloprevotella tannerae*, *Clostridiales bacterium*, *Prevotella nigrescens* and *T. denticola* were increased, whereas *Prevotella pallens* was decreased (Figure 2e). Compared to gingivitis, BEVs from *M. hyosynoviae* , *T. denticola* *and C. bacterium* were also increased in periodontitis (Figure 2e). Bacteria–bacteria network analysis of salivary MVs at the genus level using Cytoscape highlighted differentially expressed genera and their relative abundance (Figure 3a).

Discriminatory analysis of significantly expressed genera derived from BEVs showed that *Treponema 2* (AUC = 0.94; sensitivity 96%, specificity 55%) and *Fretibacterium* (AUC = 0.93; sensitivity 96%, specificity 70%) distinguished P from H (Figure 3b) and also differentiated P from G (*Treponema 2*, AUC = 0.83, *p* = 0.0004; sensitivity 76%, specificity 75% and *Fretibacterium*, AUC = 0.86; sensitivity 88%, specificity 75%; Figure 3c) as well as P from non‐periodontitis (H + G) (*Treponema 2*, AUC = 0.89; sensitivity 92%, specificity 72% and *Fretibacterium*, AUC = 0.9; sensitivity 80%, specificity 83%; Figure 3d).

At the species level, BEVs from *P. pallens* distinguished G from H controls (AUC = 0.67; sensitivity 56%, specificity 40%; Figure S5d). BEVs from *T. denticola* (AUC 0.89; sensitivity 88%, specificity 60%), *M. hyosynoviae* (AUC 0.88; sensitivity 92%, specificity 63%) and *Clostridiales* bacterium (AUC 0.8; sensitivity 80%, specificity 63%) distinguished P from H (Figure 3e). For P versus G, the AUCs were 0.83, 0.81 and 0.77, respectively (Figure 3f), and for P versus non‐P, they were 0.85, 0.85 and 0.79, respectively (Figure 3g).

### 3.4 Host DNA Methylome of Salivary MVs Distinguishes Periodontitis From Periodontally Healthy Subjects

Global DNA methylation showed a significant increase in 5mC in salivary MVs in the P group compared to the H group (Figure S6a). Further analysis identified over 150,000 methylated sites across 9 H, 12 G and 11 P individuals (Figure S6c) in various genomic regions (5′ UTR, promoter, 3′ UTR, CDS, intron and intergenic regions).

**FIGURE 4:**
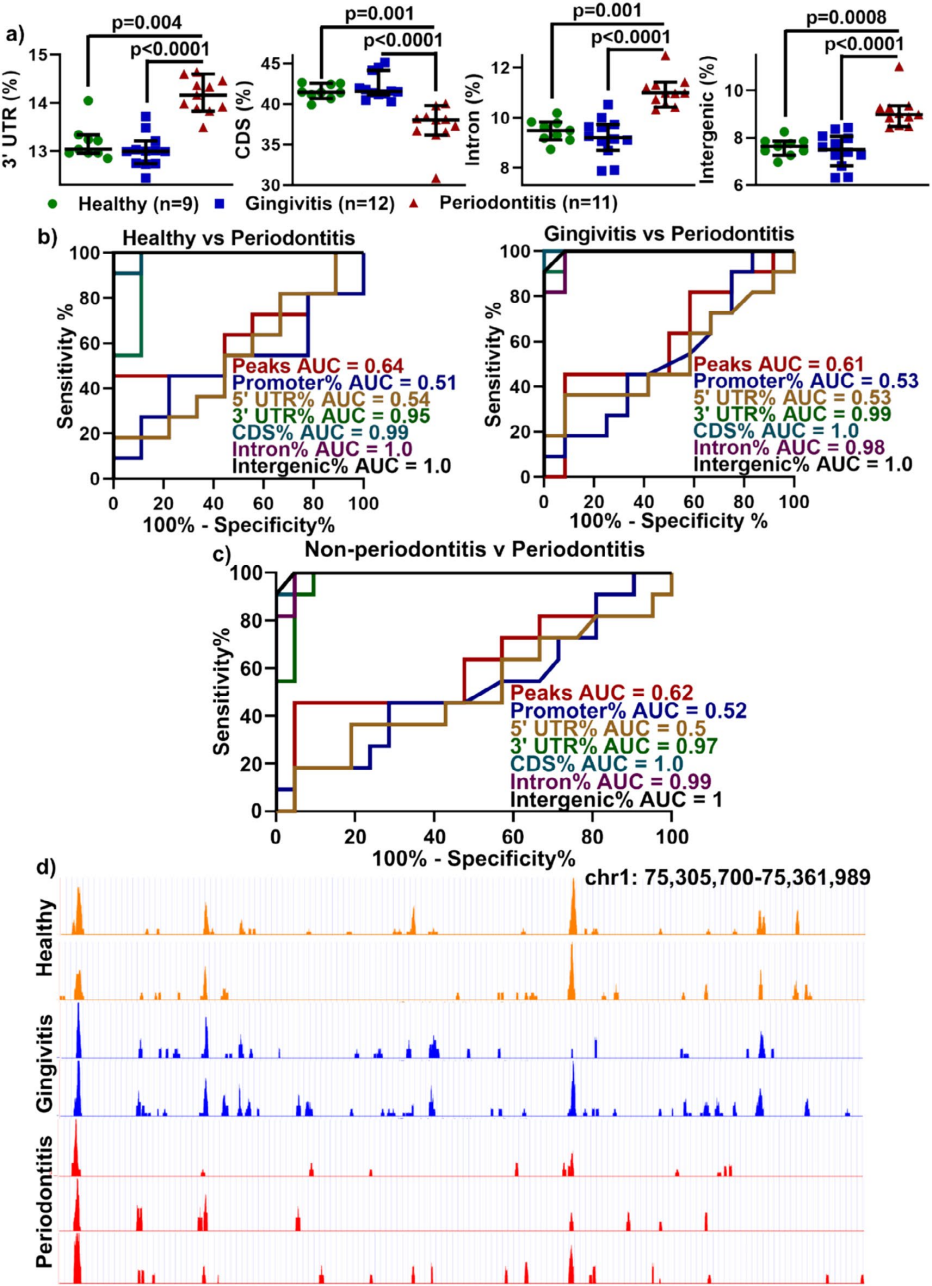
Global methylcytosine in salivary MVs as a marker for distinguishing periodontitis from non‐periodontitis patients. (a) Scatter plots of differences in global 3′ UTR, CDS, intron and intergenic 5mC in human salivary MVs. Data are displayed as scatter dot plots with a median + 95% CI. *p*‐values are from the Kruskal–Wallis test with Dunn's multiple comparison test. CDS: coding DNA sequence. (b, c) ROC curve and area under the curve (AUC) analysis for indicated parameters in periodontitis patients compared to healthy or gingivitis donors (b) or non‐periodontitis (combining healthy and gingivitis) (c). (d) Brower representation of 5mC abundance in salivary MVs for two healthy, two gingivitis and three periodontitis patients. chr1: chromosome 1.

As for MeDIP‐seq data (Figure S6b) after normalising the length of each region to 100 million base pairs for comparing the number of peaks, the global 5mC methylome of the promoter (%) and 5′ UTR (%) showed no significant difference between the groups (Figure S6d) but a significant increase in 3′ UTR (%), intron (%) and intergenic (%) regions and decreased methylation in CDS (%) regions between P and H or G groups (Figure 4a). The ROC curves and AUC values showed that the global epigenome was unable to differentiate the G group from the H group (Figure S6d).

Furthermore, the 3′ UTR, CDS, intron and intergenic regions of global 5mC epigenome profiles performed the best in stratifying P from either H, G or non‐P patients, with AUC values ranging from 0.95 to 1.0 and a sensitivity of 100% (Figure 4b,c). Abundant representative methylation sites between the three groups revealed consistency among individuals in chromosome 1 (Figure 4d), chromosome 19 and chromosome X (Figure S7). This suggests that the global epigenome profile of salivary MVs could be considered as a diagnostic biomarker for periodontitis.

**FIGURE 5:**
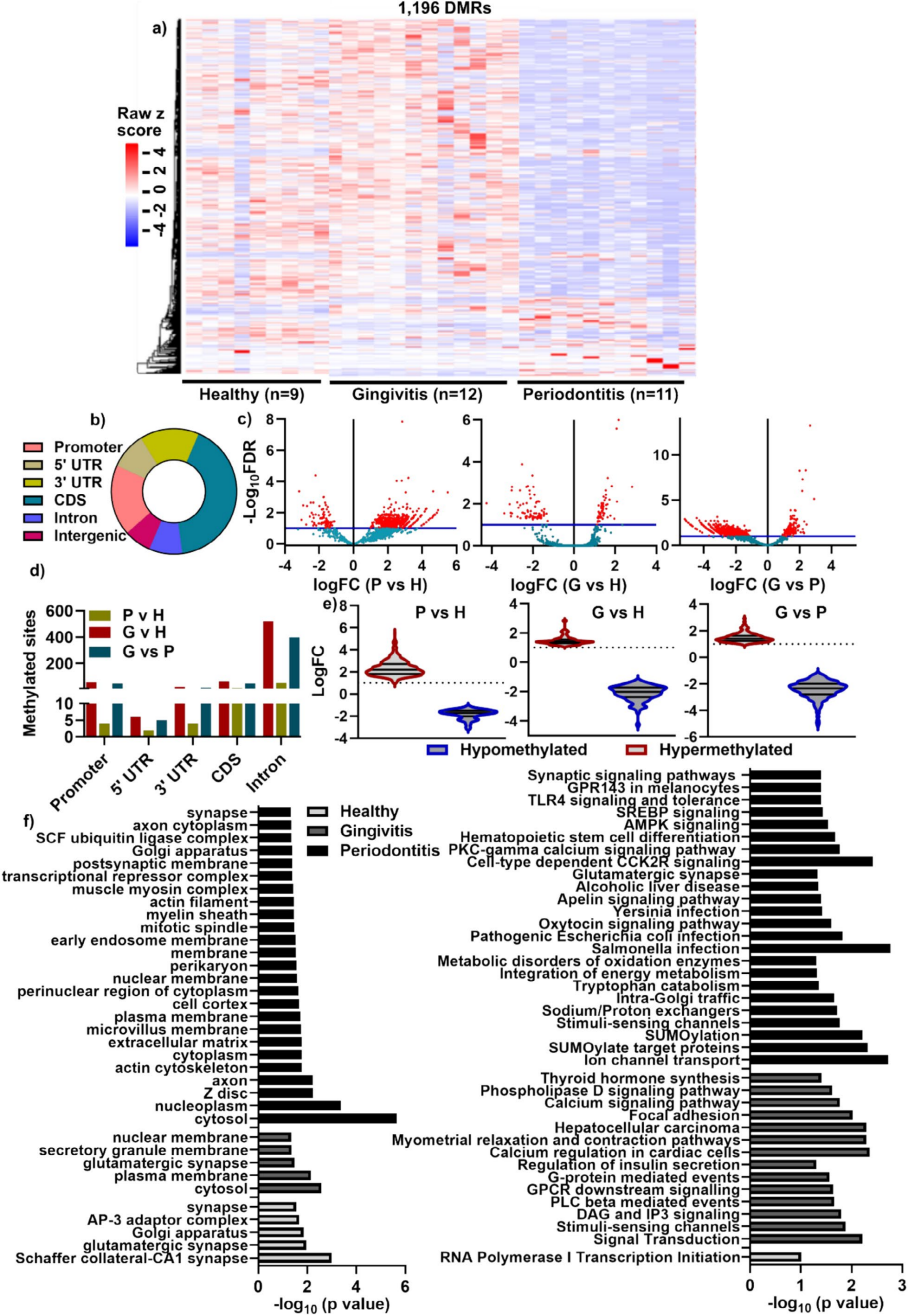
Genomic‐wide DNA methylome identifying differentially methylated regions (DMRs) in salivary MVs from periodontitis patients. (a) Heatmap of 1196 DMRs identified between 9 healthy (H), 12 gingivitis (G) and 11 periodontitis (P) derived salivary MVs. (b) Genomic‐wide 5mC distribution after normalising the length of each region to 100 million base pairs for comparing the number of peaks. Promoter region: 2000 (bp) upstream region of the gene's first exon (e.g., if the TSS position for a gene is +1, then the promoter region for this gene will be −1999 ~0). (c) Volcano plot of DMRs from P versus H, G versus H and G versus P. Red dots indicate windows significant at −log10(FDR) > 1.0. (d) Detailed 5mC DMR site distribution after group comparisons. (e) Violet graph of hypomethylated sites in periodontitis (left) and gingivitis compared with healthy (middle) and gingivitis (right). (f) Gene ontology (left) and pathways (right) analysis for gene clusters enriched in upregulated and differentially methylated genes associated with 5mC deposition in healthy, gingivitis and periodontitis groups using DAVID bioinformatics database with *p*‐values < 0.05.

To investigate whether salivary MVs MeDIP‐seq data could identify periodontitis patients, we generated DMRs between the groups at 5% FDR. We identified 1196 DMRs between the H, G and P groups (Figure 5a) and observed 5mC occupancy at the CDS and promoter regions, 5′ UTR and 3′ UTR, intron and intergenic regions (Figure 5b). At a significance level of Benjamini–Hochberf FDR of 0.1, volcano plot of DMRs (Figure 5c) and detailed 5mC DMRs showed 971 hypermethylated sites in H (vs. P), 225 in P (vs H), 657 in G (vs. H) and 1000 in G (vs. P) (Figure 5d). Further analysis showed that the majority of the 1196 DMRs were detected in the intron regions, with the least intron methylated sites after P versus H analysis (Figure 5d). Violet graph of hypomethylated sites was consistent in the periodontitis and healthy patients (Figure 5e), indicating that MeDIP‐seq is a more reliable method to uncover more methylome profiling.

Gene ontology (GO) analysis in P and G showed significant MV‐related clusters compared to H, including ‘plasma membrane’, ‘early endosome membrane’ and ‘nuclear membrane’ (Figure 5f). Pathways in periodontitis showed an enriched ion channel transport, the AMP‐activated protein kinase (AMPK) and Toll‐like receptor 4 (TLR4) pathways (Figure 5f).

### 3.5 Molecular Correlation Across Salivary MV Multi‐Omes

We analysed the correlations between the microbiome, host global methylome and MV CD markers, identifying over 40 coefficients (*r* > 0.5; Figure S8), with 14 associations in H, 16 in G and 12 in P between microorganisms and host epigenome signatures (Figures S9 and S10). In periodontitis, CD63, CD24 and CD45 positively correlated with *Campylobacter* (Figure S10). PCA showed that PC1 and PC2 accounted for 26.98% and 17.2% of variation, respectively (Figure S10). Notably, *Butyrivibrio 2* exhibited a positive correlation with CD45 MVs, sites ≥ 5 mm, methylation peak counts, intergenic region methylation and probing index.

## 4 Discussion

The pathogenesis of periodontitis involves oral microbiome dysbiosis and host methylome alterations, driving chronic inflammation and tissue destruction (Van Dyke et al. 2020). Our study provides the first proof‐of‐concept analysis that elevated CD63^+^, CD45^+^, CD29^+^ and CD24^+^ MVs, along with bacterial EVs from *Treponema*, *Fretibacterium* and *T. denticola* , could distinguish periodontitis from healthy and gingivitis cases. Genome‐wide differentially methylated regions across 3′ UTRs, CDS, introns and intergenic regions (AUC 0.95–1.0; 100% sensitivity), enriched in AMPK and TLR4 pathways, highlight the role of salivary MVs in host–microbiome interactions and their potential as real‐time diagnostic biomarkers.

Salivary MVs exhibited heterogeneous tetraspanin expressions, with CD63 demonstrating higher abundance than CD9 and CD81, consistent with established EV characterisation studies (Mathieu et al. 2021), and elevated CD9+, CD63+ and CD81+ salivary EVs reported in periodontitis (Chaparro Padilla et al. 2020; Liu et al. 2023). Our salivary MVs (16 k isolation) were around 130–150 nm, in contrast to previously reported salivary MVs (50 k isolation), peaking at 520 nm (Zhong et al. 2019). Paradoxically, the P group had fewer salivary MV particles than the H and G groups, contrasting with prior increased salivary sEVs in periodontitis (Han, Bartold, et al. 2020; Han et al. 2021; Han, Lai, et al. 2020). This difference suggests that centrifugation critically influences the size distribution and physical properties of isolated MVs and that MVs and sEVs may contain various cargo and particle numbers in periodontitis. Despite the reduced MV particle numbers, the P group showed enriched CD40+, CD63+, CD31+, CD29+, CD45+ and CD24+ MV sub‐populations, reflecting an enhanced vesicles release (CD63+) (Joshi et al. 2020) involved in immune activation (CD40+, CD45+) (Dutzan et al. 2012), endothelial damage (CD31+) (Yılmaz Şaştım et al. 2021) and altered tissue remodelling of cell–matrix interactions (CD29+), characteristic of chronic periodontal inflammation and tissue destruction in periodontitis (Kasprzak et al. 2012). These findings warrant further investigation on the specific biological roles of MVs in periodontitis and their potential as diagnostic biomarkers.

Bacteria‐derived EVs (BEVs) derived from dental biofilm are likely the key pathogenic drivers that shed from the biofilm and can diffuse into the periodontal tissues (Liu et al. 2025; Ma and Cao 2021). Understanding the role of BEVs in the pathogenesis of periodontitis is essential for developing new diagnostics, particularly gram‐negative bacteria–derived outer membrane vesicles (OMVs) (Bartold and Van Dyke 2019). Previously, we and others demonstrated that bacterial DNA or lipopolysaccharide (LPS+) OMVs were found in saliva (Han, Lai, et al. 2020; Park et al. 2021) and BEV proteins were found in GCF‐derived EVs (Mizgier et al. 2025). However, limited studies have explored the microbial components of salivary MVs in periodontitis. Our finding is that genus *Treponema 2* and *Fretibacterium‐*enriched BEVs, particularly *T. denticola–*derived BEVs, can differentiate P from G (AUC > 0.9), consistent with the previous studies identifying these genera and species in subgingival biofilm (Oliveira et al. 2016) and plaque (Zeng et al. 2021), indicating that salivary OMVs may reflect subgingival plaque microbiota. Importantly, *T. denticola–*derived OMVs contain msRNA and outer membrane proteins (Choi et al. 2017; Veith et al. 2019), which can drive naïve CD4+ T cells towards Th1 or Th17 cells (Lim et al. 2022) and enhance TNFα, IL‐8 and IL‐1β cytokine secretion in monocytes (Cecil et al. 2017). *Treponema 2* plays a crucial role in interacting with CD24+ epithelial cells during periodontitis progression (Tribble and Lamont 2010). These immunomodulatory properties of BEVs highlight their potential as biomarkers, reflecting both microbial dysbiosis and host immune activation in periodontitis. However, distinguishing from host EVs and gram negative bacteria–derived OMVs remains challenging since no universal OMV surface markers are available. An alternative approach could be to use immunoaffinity techniques to deplete host EVs (Liu et al. 2023), thereby enriching OMVs.

DNA methylation is dynamically modulated by environmental factors and contributes to the pathogenesis of many complex diseases (Khouly et al. 2020). In periodontitis, recent studies have examined promoter‐specific gene methylation changes in periodontal tissues (Zhang et al. 2010; Ishida et al. 2012; Asa'ad et al. 2017) or salivary sEVs (Han, Lai, et al. 2020), suggesting their potential as biomarkers. Recent studies suggest that periodontal inflammation and tissue destruction are linked to significant changes in methylation, which may regulate gene expression and disease progression (Zhao et al. 2022). However, little is known about the genome‐wide DNA methylome landscape of salivary MVs. In our study, a significant increase in 5mC global DNA methylation (using ELISA) was observed in periodontitis salivary MVs, consistent with our previous observations (Han et al. 2021). Furthermore, we employed low‐input and highly sensitive MeDIP‐seq to capture 5mC profiles from individual salivary MV samples (Han et al. 2023). The 3′ UTR, CDS, intron and intergenic epigenome profiles effectively distinguish periodontitis from healthy and gingivitis patients, with AUCs of 0.95 to 1.0 and 100% sensitivity, highlighting their potential as reliable epigenetic diagnostic biomarkers. Beyond diagnosis, pathway enrichment analysis revealed hypermethylation in genes associated with ion channel transport, AMPK and TLR4 pathways (Figure 5f), aligning with prior reports of AMPK signalling (Tong et al. 2020; Ren et al. 2021) and TLR4 pathways (Song et al. 2017) in periodontitis, linking microbial recognition with host immune and metabolic responses for periodontitis. Our profiling of both DNA methylome and microbiome advances our understanding of the characteristics of EVs in periodontal health and disease, supporting the goal of precision periodontics to define diseases based on molecular mechanisms and pathophysiology (Lam et al. 2021; Cheng and Hill 2022).

This exploratory clinical study has several limitations, including a relatively small cohort size, lack of control for toothpaste uses prior to sampling, absence of metagenomic sequencing, no comparison with other biofluid sources (i.e., plasma), the use of a mixture of host‐derived EVs and bacterial BEVs following 16,000*g* centrifugation and its cross‐sectional design. Despite these limitations, our study is the first to demonstrate that salivary MVs in periodontitis originate from both immune cells and gram‐negative bacteria (*Treponema 2* and *Fretibacterium*), highlighting their dual host and microbial origin and extending previous evidence that sEVs from GCF carry both host and bacterial proteins. Although our findings suggest that both salivary host‐derived EVs and bacterial OMVs have potential diagnostic value, their specificity and sensitivity have not been fully established. Further validation in larger prospective longitudinal studies will be essential to assess the diagnostic performance and clinical applicability of both methylation and microbial profiles.

**FIGURE 6:**
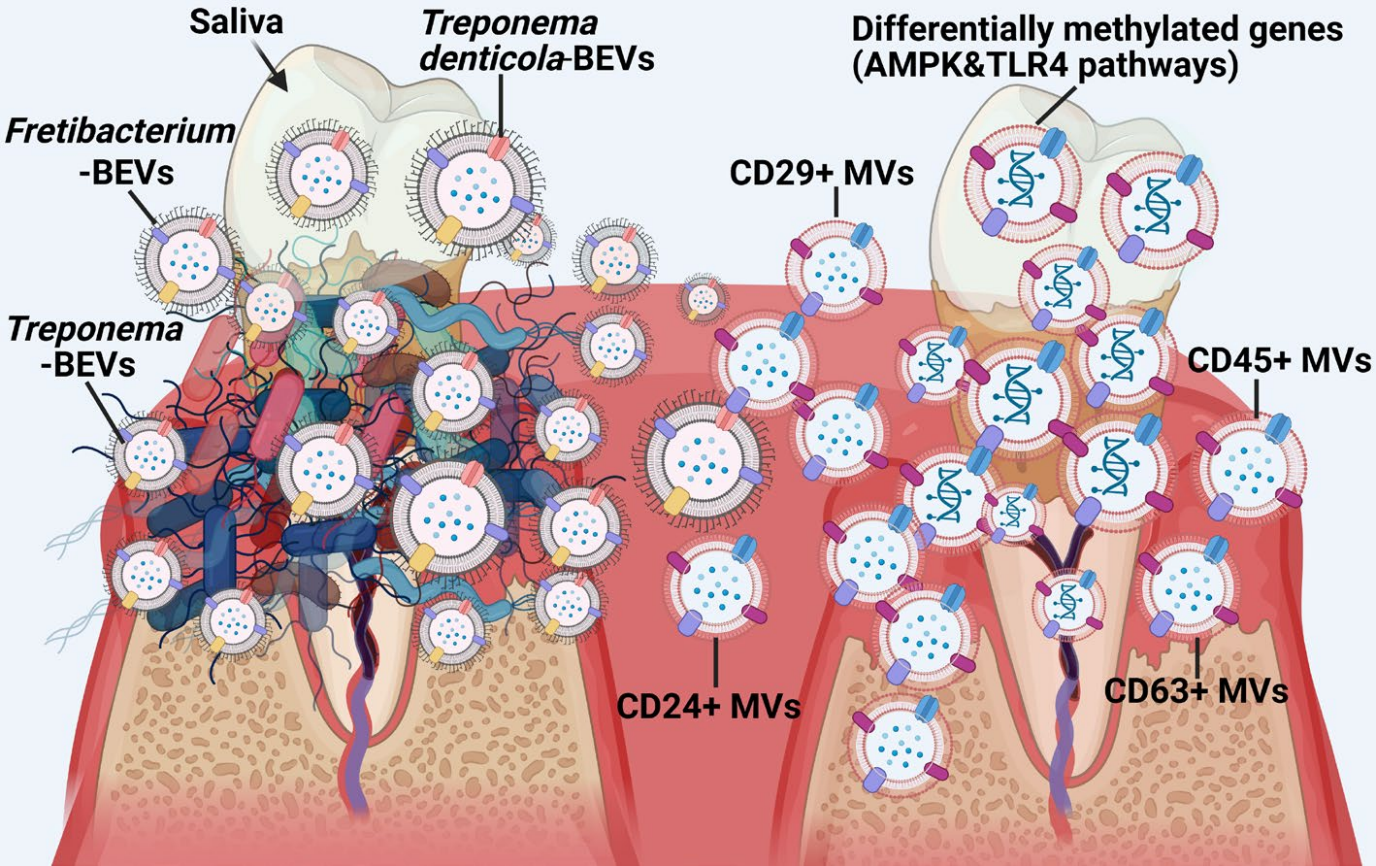
Conclusion of this exploratory study: Salivary microvesicles (MVs) revealed host methylome and microbiome biomarkers of periodontitis, including enriched CD63u207a, CD45u207a, CD29u207a, and CD24u207a MVs subpopulations; bacterial extracellular vesicles from Treponema, Fretibacterium, and T. denticola; and differential host DNA methylation in genes linked to inflammation‐related pathways (AMP‐activated protein kinase, AMPK and Toll‐Like Receptor 4, TLR4).

## 5 Conclusion

Salivary MVs, both host‐ and microbial‐derived, reflect periodontal disease status, with increases in CD45^+^, CD29^+^ and CD24^+^ host MVs subpopulations, bacterial OMVs from *Treponema*, *Fretibacterium* and *T. denticola* as well as higher percentages of genome‐wide methylated regions across 3′ UTRs, CDS, introns and intergenic regions found in periodontitis patients (Figure 6). These exploratory findings highlight the potential of salivary MVs as non‐invasive biomarkers for periodontitis, warranting validation in larger longitudinal studies.

## Author Contributions

P.H. wrote the main manuscript, performed most of the experiments, prepared all the figures, attracted funding and edited the manuscript. C.S., Q.Z., C.S., X.L. and S.I. wrote parts of the manuscript, edited the manuscript and assisted with the figure and method optimisation. All authors approved the final version of the manuscript.

## Funding

This work was supported by the Australian Dental Research Foundation (534‐2019), National Health and Medical Research Council (2034591) and National Natural Science Foundation of China (82001421).

## Conflicts of Interest

The authors declare no conflicts of interest.

## Supporting information


**Data S1:** The detailed methodology and additional data supporting the findings of this study are available in Figures S1–S10. The raw omics files are available from the corresponding author upon reasonable request.

## Data Availability

The data that support the findings of this study are available in the Supporting Information of this article.
