# Supplementary material for: Salivary Microvesicle Methylome and Microbiome Profiles in Periodontitis: An Exploratory Study
Source: J Clin Periodontol. 2026 Mar 26;53(6):911–23. doi: 10.1111/jcpe.70114 (PMC13167645; doi:10.1111/jcpe.70114)
Supplement: Supplementary file 1 — Data S1: The detailed methodology and additional data supporting the findings of this study are available in Figures S1–S10. The raw omics files are available from the corresponding author upon reasonable request. [file JCPE-53-911-s001.docx]

**Supplementary information for**

**Salivary microvesicle methylome and microbiome profiles in periodontitis: an exploratory study**

Pingping Han^1,2,*^, Chaminda Seneviratne^1^, Qiongyi Zhao^3^, Carlos Salomon^4^, Xiang Li^5,*^, Sašo Ivanovski ^1,2,*^

1. The University of Queensland, School of Dentistry, Brisbane, QLD 4006, Australia
2. The University of Queensland, School of Dentistry, Center for Oral-facial Regeneration, Rehabilitation and Reconstruction (COR3), Epigenetics nanodiagnostic and therapeutic group, Brisbane, QLD 4006, Australia
3. The University of Queensland, Queensland Brain Institute, Brisbane, 4072, Australia
4. The University of Queensland, Faculty of Medicine, Translational Extracellular Vesicles in Obstetrics and Gynae-Oncology Group, Centre for Clinical Diagnostics, University of Queensland Centre for Clinical Research, Royal Brisbane and Women’s Hospital, Brisbane, QLD, 4029, Australia
5. Department of Anesthesiology, Brain Research Center, Department of Neurosurgery, Zhongnan Hospital, Wuhan University, Wuhan, Hubei Province, China.

*Corresponding authors:

Pingping Han: [p.han@uq.edu.au](mailto:p.han@uq.edu.au)

Xiang Li: [li.xiang@whu.edu.cn](mailto:li.xiang@whu.edu.cn)

Sašo Ivanovski: [s.ivanovski@uq.edu.au](mailto:s.ivanovski@uq.edu.au)

**Materials and Results**

**Patient recruitment**

The screening inclusion criteria were: ≥ 18 years old; no self-reported systemic diseases; and ≥ 20 teeth (excluding third molars). Exclusion criteria include immunosuppression, antibiotic medicine for over three months, uncontrolled medical conditions; and over 6 months of use of anti-inflammatory medications.
All participants reported being non-smokers and did not indicate the presence of any systemic diseases. There was no prior power analysis performed to determine the sample size before this project commenced. Study groups were allocated according to our previously published clinical parameters [1, 2] and the latest periodontal disease classification guidelines [3] (a) The Healthy (n=20) group contained individuals with no previous history of periodontal disease, probing pocket depth (PPD) less than 3 mm, and bleeding on probing (BOP) at equal to or less than 10% of sites. (b) The gingivitis group (n=16) comprised individuals who did not exhibit deep periodontal pockets (PPD < 3 mm) but had more than 10% of sites with BOP. (c) The stage III/IV periodontitis group (n=26) included individuals with periodontal disease characterized by greater than 30% of sites displaying ≥3 mm PPD along with BOP. Additionally, they exhibited PPD of 5 mm or greater at over five sites across at least three non-adjacent teeth.

**Salivary microvesicles isolation and characterisation**

Unstimulated whole saliva was collected from each participant as in our previous published protocols [17, 18]. Participants abstained from consuming food and beverages for at least one hour before saliva collection. Each participant was given 15mL of water to wash their mouth and eliminate any food debris. After spitting the saliva into a sterile falcon tube, saliva samples were placed on ice and then kept at -80°C. Following saliva collection, a full-mouth periodontal chart was performed by registered periodontists to determine periodontal health status. Participant screening was carried out according to periodontal health guidelines and followed the chart in Supplementary Figure 1.

Saliva microvesicles (MVs) were isolated using serial centrifugation as previously described [2, 4]. Briefly, 500 μL of saliva was diluted with 500 μL 1x phosphate-buffered saline (PBS, calcium-, magnesium-, phenol red-; ThermoFisher). The samples underwent sequential centrifugation steps at 4°C: first, centrifugation at 300 g for 15 minutes removed cells, followed by 2,600 g for 15 minutes to eliminate apoptotic bodies. The subsequent supernatant went for centrifugation at 16,000 g for 20 minutes to enrich MVs. Upon resuspension in 500 μL of PBS, MVs underwent characterization following the guidelines from the Minimal Information for Studies of Extracellular Vesicles 2018 (MISEV2018) [5]. The characterization of MVs involved assessing their morphology using transmission electron microscopy (TEM), identifying EV surface markers using the human MACSPlex Exosome Kit, determining particle number and size through nanoparticle tracking analysis (NTA), and employing Fourier Transform Infrared spectroscopy (FTIR).

The TEM analysis was carried out in accordance with the previously established protocol [1]. Briefly, MVs were initially fixed in 3% glutaraldehyde and then attached onto electron microscopy grids coated with formvar carbon. After three rinses with phosphate-buffered saline (PBS), these grids were immersed in a uranyloxalate solution (pH=7) for 3 mins, and finally, visualised on an FEI Tecnai T12 Transmission Electron Microscope (FEI, Hillsboro, USA).

Salivary MVs were labelled by 25 μg/mL of 3, 3′-Dioctadecyloxacarbocyanine perchlorate (DiO, Sigma) at RT for 1 h. Excess, unbounded DiO was eliminated at 4,000× g for 5 min at 4 °C using an Amicon Ultra 0.5 Centrifugal Filter Unit (10 kDa, Merck Millipore). The DiO-labelled MVs (green) were subsequently visualized using a Nikon Confocal Microscope (Nikon, Tokyo, Japan).

Following the manufacturer’s protocol, the protein quantification of MVs was assessed using a Pierce™ BCA Protein Assay Kit (ThermoFisher Scientific). MVs purity was determined as EVs particle number per µg protein.

To determine the MVs particle numbers and size, the NTA analysis was conducted using a NanoSight NS500 instrument (NanoSight, Salisbury, UK), which was equipped with a 488 nm laser and NTA 3.1 software. As reference materials, 100 nm polystyrene latex beads (Malvern NTA 4088) and 1x PBS were used as positive and negative controls, respectively. Five 30-second videos for each sample were obtained with a camera level of 14 and a detection threshold of 5. Subsequently, the video data underwent processing and analysis to determine both particle size and particle concentration.

The biochemical components of saliva MVs were measured by FTIR measurements with a Nicolet™ iS20 FTIR Spectrometer (Thermo Scientific™) in transmission mode as described previously [6, 7]. 2µl of MVs sample was deposited onto a diamond probe before obtaining the measurements ranging from 600-4000 cm^-1^, with 64 scans per sample. PBS was used as a background to subtract from.

**Host MVs surface signature using a multiplex platform**

The analysis of 37 surface markers on extracellular vesicles (EVs) and distinct subpopulations of MVs was conducted with a human MACSPlex Exosome Kit (Miltenyi Biotec, Bergisch-Gladbach, Germany) as per the manufacturer's instructions and described previously [8, 9]. 5 µg of MVs were mixed with 15 μL MACSPlex Exosome Capture Beads in MACSPlex buffer before being incubated at 4^o^C overnight. Then, after 3000× *g* for 5 min at 4^o^C, the samples were incubated with 5 μL MACSPlex Exosome Detection Reagent at RT for 1 hour. After washing with MACSPlex buffer, MV-containing samples were subjected to a BD FACSVerse™ Flow Cytometer (BD, Becton, Dickinson U.K. Limited). The median fluorescence intensity (MFI) values were quantified using FlowJo software (V11, FlowJo LLC) for all 37 EVs surface epitopes capture bead subsets after. subtracting the background signal from mIgG isotype control. The scatter plot graph with the individual subject was created using Prism Graphpad 10. The differentially expressed CDs markers interaction between proteins was generated using the STRING network [10], which covers the known and predicted protein-protein network.

**Salivary MVs DNA isolation**

Prior to DNA isolation, MVs were pre-treated in 2% Triton X-100 for 10 mins at 55^o^C and then lysed in 0.2% SDS, 5 mM EDTA, 100mM Tris-HCl (pH 8.0), 200mM NaCl, 300 µg/mL proteinase K and 300 µg/mL of RNase A in PBS for overnight at 55^o^C. For each sample, an equal volume of phenol:cholorform:isoamyl alcohol (in a ratio of 25:24:1, ThermoFisher Scientific) was added, followed by vortexing for 2 minutes. Following this step, the samples underwent centrifugation at 14,000 g for 5 minutes at RT and the supernatant was combined with 2.5 times its volume of ice-cold 100% molecular-grade ethanol (ThermoFisher Scientific), along with 1 µl of glycogen (20 mg/ml; ThermoFisher Scientific) and NaCl (a final concentration of 250 mM).

DNA was allowed to be precipitated for 4 h at −30°C and centrifuged at 15,000 g for 30 min at 4°C. The resulting DNA pellets were washed with ice-cold 70% ethanol and further centrifuged at 15,000 g for 10 minutes at 4°C. After removing the ethanol, the DNA pellets were air-dried before being resuspended in 25 µl of ultrapure H_2_O. To maximize the DNA recovery, the solution was heated to 37°C in a thermomixer for 30 minutes. Quantification and assessment of DNA quality were carried out using the Qubit dsDNA HS assay (Invitrogen) and Nanodrop (Thermofisher, Waltham, MA, USA). Additionally, DNA quality was further evaluated using the DNA High Sensitivity Chip on an Agilent 2100 bioanalyzer (Agilent Technologies).

**MVs microbiome by 16S rRNA sequencing**

The microbiota within salivary microvesicles (MVs) was characterised by employing DNA sequencing targeting the V3 and V4 hypervariable regions of the 16S rRNA gene. This sequencing was conducted using an Illumina MiSeq instrument (Illumina, San Diego, CA, USA) in conjunction with proprietary primers designed by GENEWIZ. For this analysis, 2-20 nanograms of MVs DNA were utilised to generate amplicons featuring an associated linker with an index. Subsequently, the DNA library was subjected to purification using AMPure XP magnetic beads (Beckman Coulter), and its concentration was determined via Nanodrop and Agilent 2100 Bioanalyzer system. The library was then quantified to 10 nM and subjected to paired-end sequencing with a read length of 300 bases (FE300) on the Illumina MiSeq platform, following the guidelines specified in the instrument manual. The sequencing utilised SBS reagents v2 for MiSeq. Data analysis and control of the MiSeq instrument were executed using Novaseq Control Software (NCS) to ensure accurate and reliable reads.

The sequencing data obtained from Illumina double-end sequencing, encompassing both positive and negative reads, were subjected to a series of data processing steps. Initially, the sequences were merged, and those containing ambiguous bases (N) were removed. Only sequences with a length exceeding 200 base pairs were retained following a quality filter. Subsequently, chimeric sequences were eliminated from the dataset. The remaining sequences were clustered into operational taxonomic units (OTUs) at a 97% sequence similarity using the VSEARCH clustering software (version 1.9.6). The reference database employed for this clustering was the Silva 132 16S rRNA reference database. Taxonomic assignment was then finalised using the Ribosomal Database Program (RDP) classifier, which utilises a Bayesian algorithm.

The dataset used in the analysis consisted of an average of 55,458 sequences per sample, with a standard deviation of 14,896 (mean ± SD; ranging from 20,929 to 86,352 sequences per sample). In total, 3,438,397 sequences were included in the analysis, resulting in the identification of 229 OTUs. To ensure fair comparisons, a rarified counts matrix was applied in this study. Alpha diversity metrics, including Chao1, Shannon, and ACE, were computed, along with beta diversity analyses using Principal Coordinate Analysis (PCoA) based on Bray–Curtis dissimilarity indexes. Differentially abundant microbial biomarkers at the genus level were identified using Metastats, with a false discovery rate (FDR) threshold set at <0.05. Detailed statistical analyses and visualisation of diversity metrics were performed using R.

**Global DNA methylation in salivary MVs**

We conducted an analysis of global DNA methylation, including 5-methylcytosine (5mC), 5-hydroxymethylcytosine (5hmC), and N6-methyladenosine (m6dA), using DNA samples obtained from 20 healthy individuals, 16 individuals with gingivitis, and 26 individuals with periodontitis. This analysis was performed with a few specific kits, namely the Global DNA Methylation Assay Kit (5mC, ab233486, Abcam), Global DNA Hydroxymethylation Assay Kit (5hmc, ab233487, Abcam) and m6dA DNA Methylation Assay Kit (m6dA, ab233488, Abcam. The experiment protocol and data analyses were conducted as per the manufacturer’s instructions and a previously published protocol [2]. However, global methylation profiles do not provide information on the methylation patterns, so we employed low-input highly sensitive methylated DNA immunoprecipitation sequencing (MeDIP-seq) to capture the profile of 5mC derived from salivary MVs of individual samples.

**Sensitive DNA methylome profile using MeDIP-seq sequencing and data analysis**

Supplementary Figure 6e illustrates a schematic depiction of the MeDIP-seq protocol. The MVs DNA was sheared to ~200 bp using an M220 ultrasonicator (Covaris) and a microRUBE AFA sonification tube (Cavaris) under the following conditions: peak power of 50, duty factor of 20, cycles/Burst of 200, and a treatment duration of 150 seconds. The DNA fragmentation was then confirmed using a Bioanalyzer.

Prior to MeDIP, library preparation of fragmented DNA was carried out using a Kapa HyperPrep Kit (Kapa Biosystems), following the manufacturer’s instructions. In brief, following the end-repair and A-tailing steps, samples were ligated to 0.0375 μM of KAPA adapters (KAPA single-indexed adapter kit for Illumina platform) through incubation for 15 min at 20 °C, followed by purification with AMPure XP beads (Beckman Coulter). The library was eluted and then quantified by Bioanalyser and KAPA library quantification kit (Kapa Biosystems for Illumina platform). 10% of the DNA library was used as input DNA.

To ensure sufficient DNA (100ng) for MeDIP, 12 prepared libraries (22nM each) were pooled together and subjected to MeDIP with Active Motif MeDIP kit (55009) following the manufacturer’s instructions. In brief, pooled libraries were heat-treated at 95 °C for 10 min and instantly transferred to ice for another 10 minutes. Immunoprecipitation was conducted on each sample using a 5-mC monoclonal antibody and a bridging antibody from the MeDIP kit. Next, Protein G magnetic beads were added and incubated at 4 °C overnight. The samples were purified using AMPure XP beads (Beckman Coulter) and eluted in 30 μl of Neutralization buffer. After quantification with Qubit, MeDIP samples were amplified using Kapa HiFi Hotstart Mastermix and KAPA library amplification primer mix. The final libraries underwent amplification with the following cycles: 98 °C for 45 seconds, 19 cycles of 98 °C for 15 seconds, 60 °C for 15 seconds, and 72 °C for 30 seconds, with a final extension at 72 °C for 1 minute. The amplified libraries were further purified using AMPure XP beads (Beckman Coulter) and eluted to ultrapure water. All the final libraries were determined by Bioanalyzer and Qubit prior to being sequenced on an Illumina Hiseq4000 at GENEWIZ. The sequencing was carried out with a single read of 150 base pairs, multiplexing 12 samples per lane.

After the sequencing step, we utilized cutadapt version 1.17 (available at https://cutadapt.readthedocs.io/en/stable/) to remove nucleotides with low quality (Phred quality score < 20) and to trim Illumina adapter sequences found at the 3' end of every read. Following this preprocessing step, we aligned the resulting reads to the human reference genome (hg38) using BWA version 0.7.17 with default settings [11]. The resulting SAM files from the BWA alignment were transformed into BAM format, ensuring the elimination of duplicate reads. These reads were then sorted, and indices were created using SAMtools (version 1.8). Macs2 (v2.2.4) was used to call peaks for individual samples, using “-q 0.05” as the cutoff. In each condition, peaks from multiple biological replicates were then merged using bedtools (v2.27.1) with the “multiIntersectBed” function. To ensure the consistency of the peak calling across multiple biological replicates in each group, only peaks supported by more than half of the biological replicates were used for downstream analyses. Peaks overlapping with the regions in hg38 blacklist were further removed. Thus, only 9 healthy, 12 ginvititis and 11 periodontitis were included in Figure 5. The number of properly aligned reads in each peak region was extracted for each individual sample. The global epigenome profile was then compared among the healthy, the gingivitis and the periodontitis groups using edgeR (v 3.28.1) with FDR<0.05 [12]. A commonly methylated gene network was performed using GeneMANIA software (v3.6.0). Enrichment of DMRs genes was analysed for function clusters, Gene Ontology and pathways using DAVID (v6.8) [13].

**Statistical analysis**

The data are presented as a median ± 95% confidence interval (CI) for graphs. The normality of data distribution was calculated in Prism using the D’Agostino-Pearson test with an α cut-off at 0.05and all datasets showed a non-parametric distribution. We employed a non-parametric Kruskal–Wallis test with Dunn’s multiple comparisons to assess data among healthy, gingivitis, and periodontitis patients. A significance level of p < 0.05 was applied to identify statistically significant differences.

We utilised GraphPad Prism software (San Diego, SA, USA) to create Receiver Operating Characteristic (ROC) curves and compute the Area Under the Curve (AUC) values using the Wilson/Brown method. Data from patients (gingivitis and periodontitis) and healthy controls were employed for this analysis. Sensitivity and specificity were determined when choosing cut-off values of group mean values. Pearson’s correlation was performed between clinical parameters and microbiome/CDs/methylome, as well as between microbiome and CDs or methylome. All data, including clinical parameters, age, gender, disease status, global methylation profile, CD markers and microbiota, were analysed using multivariate analysis in GraphPad Prism 10.1.

**
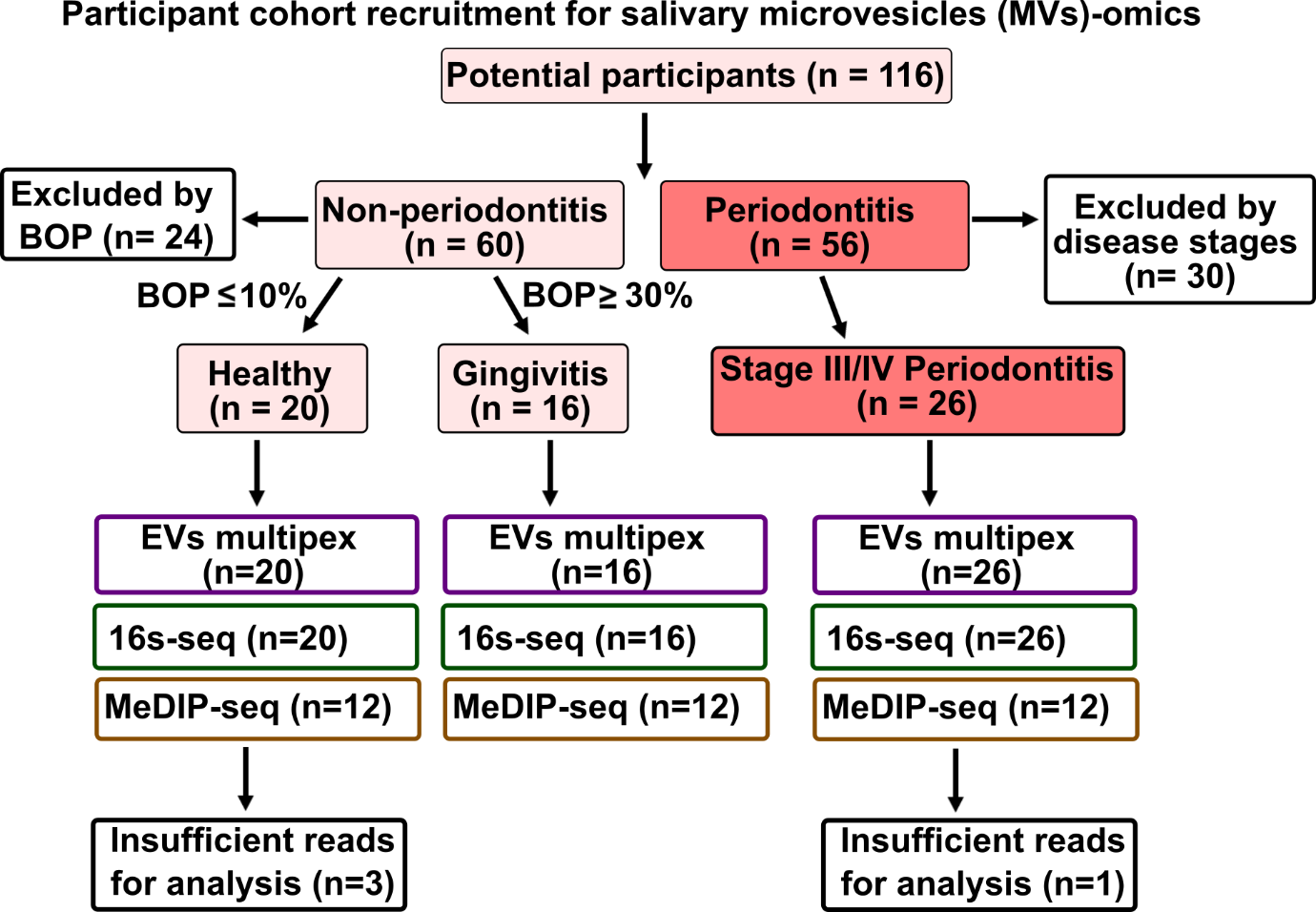
**

**Supplementary Figure 1. Participants’ cohort recruitment and downstream experiments.** BOP: bleeding on probing.

**
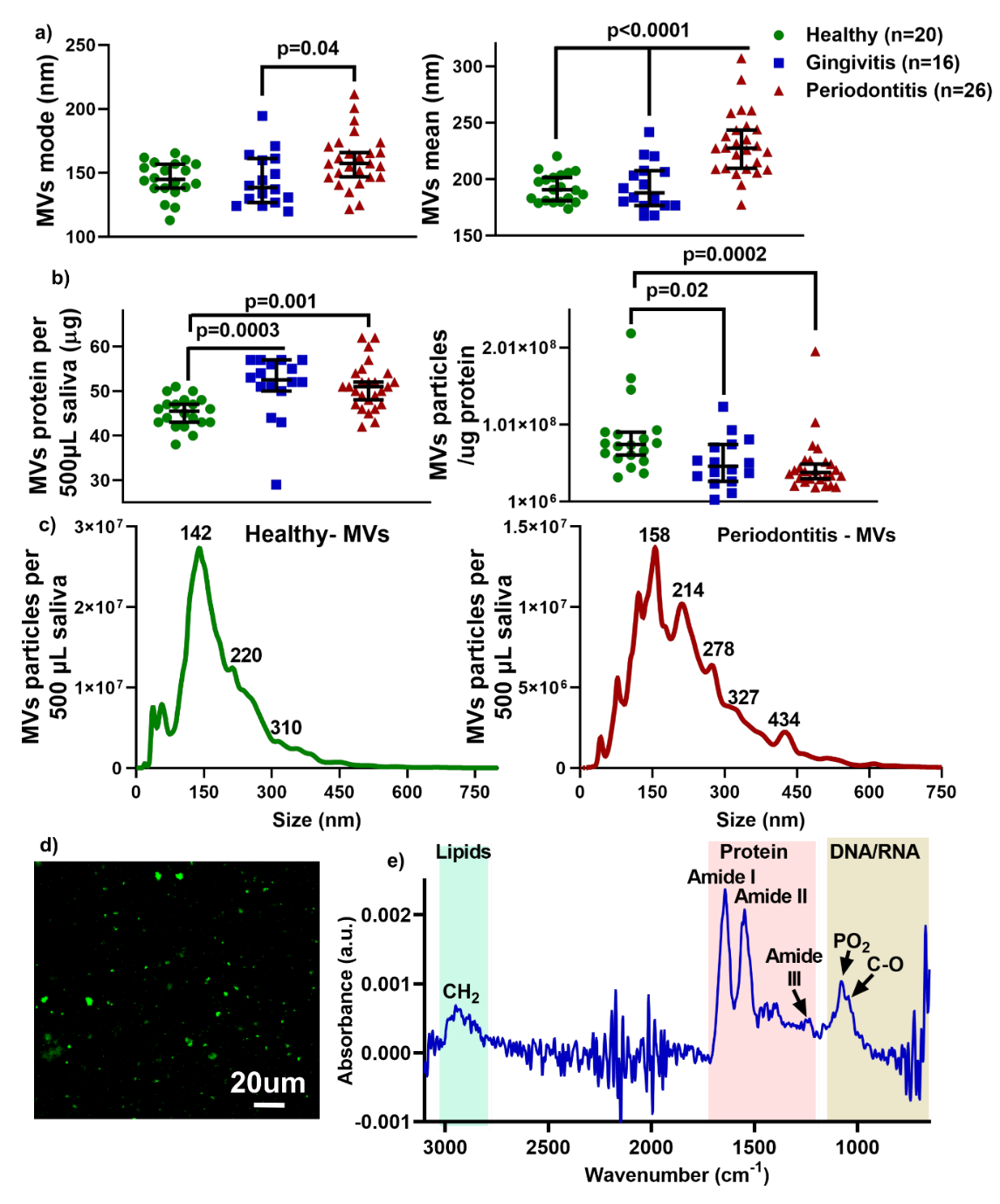
**

**Supplementary Figure 2. Characterisation of salivary MVs. a)** MVs mode and mean based on NTA data. **b)** EV protein content (left, via BCA assay) and EV particles per μg protein (right). **c**) Representative histogram of EV size distribution for healthy and periodontitis-derived MVs. **d**) Confocal microscope images of DiO-stained MVs (green). **e)** Representative FTIR spectra for salivary MVs, with peaks at protein (Amide I, II and III at 1200-1800 cm^-1^), nucleic acids (PO_2_ and C-O stretching at 960 – 1100cm^-1^) and lipids (CH_2_ stretching at 2800-3000 cm^-1^). In **a,b**: Data are displayed with scatter dot plot graphs with meadian±95% CI. P values were calculated using the Kruskal-Wallis test with Dunn's multiple comparisons test.

**
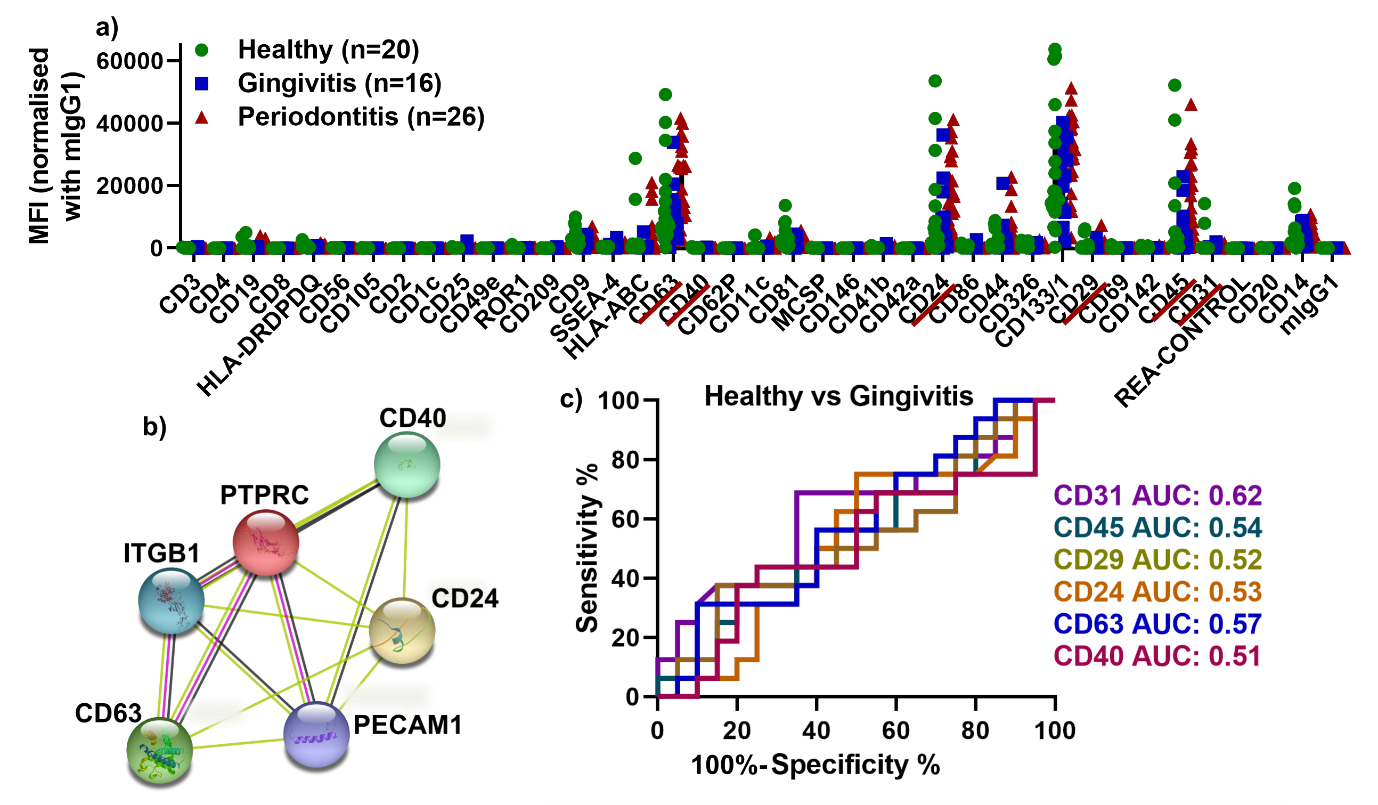
**

**Supplementary Figure 3. EV subpopulation analysis for salivary MVs using MACSPlex exosome kit. a)** Surface expression levels of 39 EV CDs markers, with median fluorescence intensity (MFI). Each dot denotes individual data from each participant. Red underlined EV subpopulations show the significantly expressed CDs markers between healthy, gingivitis and periodontitis groups. **b)** Protein interaction network between significant CDs using String software. Node colour denotes the first shell of interactions. Different colour lines showed known interaction or predicted interaction. PTPRC (protein tyrosine phosphatase receptor type C) is known as CD45. ITGB1 (integrin subunit beta 1) is known as CD29. Platelet endothelial cell adhesion molecule-1 (PECAM-1) is known as CD31. **c)** ROC curves show the discrimination power for CDs for healthy and gingivitis.

**
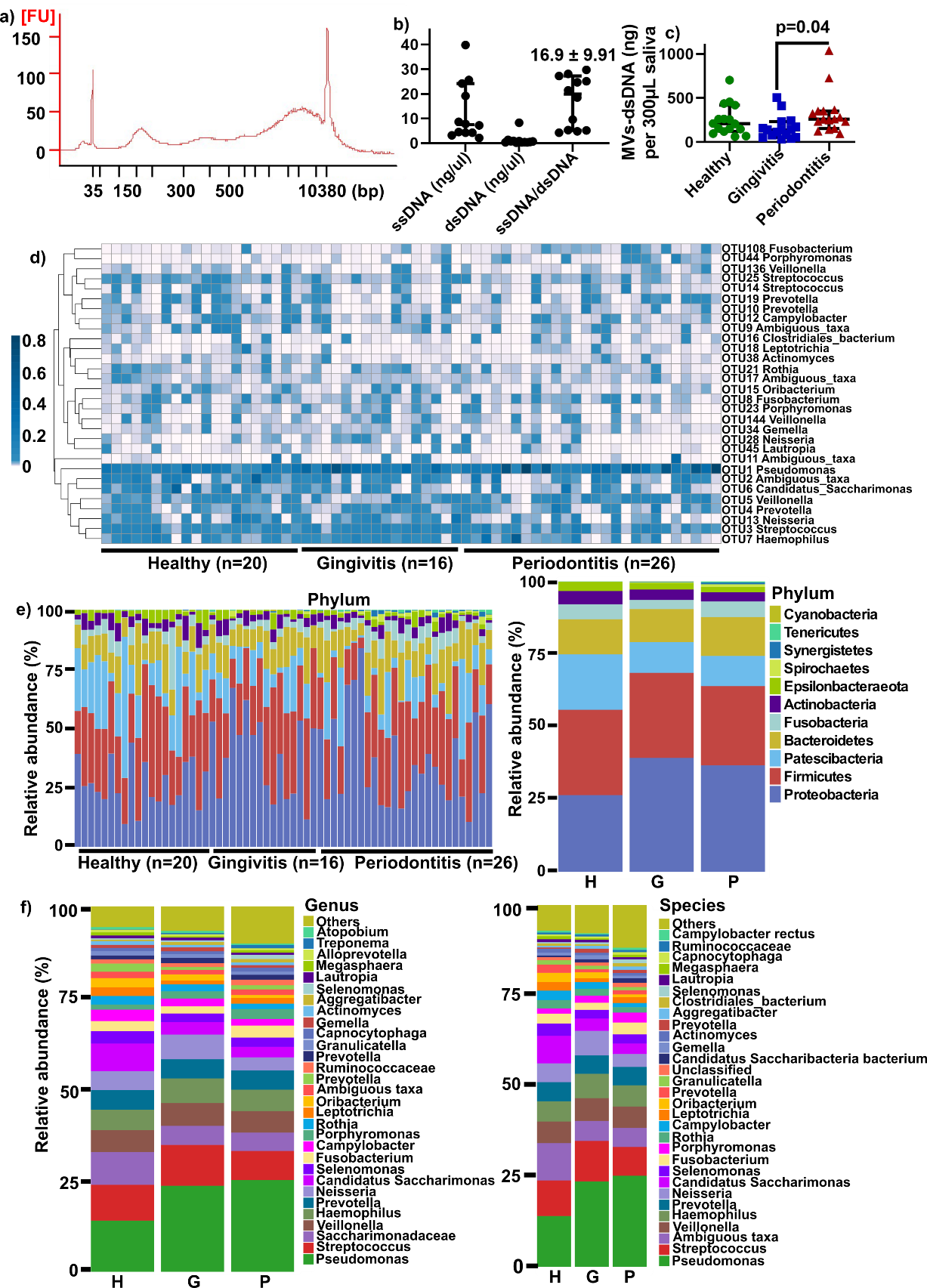
**

**Supplementary Figure 4. MVs-DNA profile, and the microbiome of salivary MVs in periodontitis. a)** Representative bioanalyser results of MV-dsDNA, indicating genomic dsDNA is present in salivary MVs. **b-c)** Qubit results of ssDNA and dsDNA (b) and total MVs-dsDNA amount. **d)** OUT abundance heatmap for healthy, gingivitis and periodontitis-derived salivary MVs. **e)** The phylum abundance for individuals (left) and grouped data (right). **f)** The Genus (left) and species (right) expression profiles between healthy (H, n=20), gingivitis (G, n=16) and periodontitis (P, n=26).

**
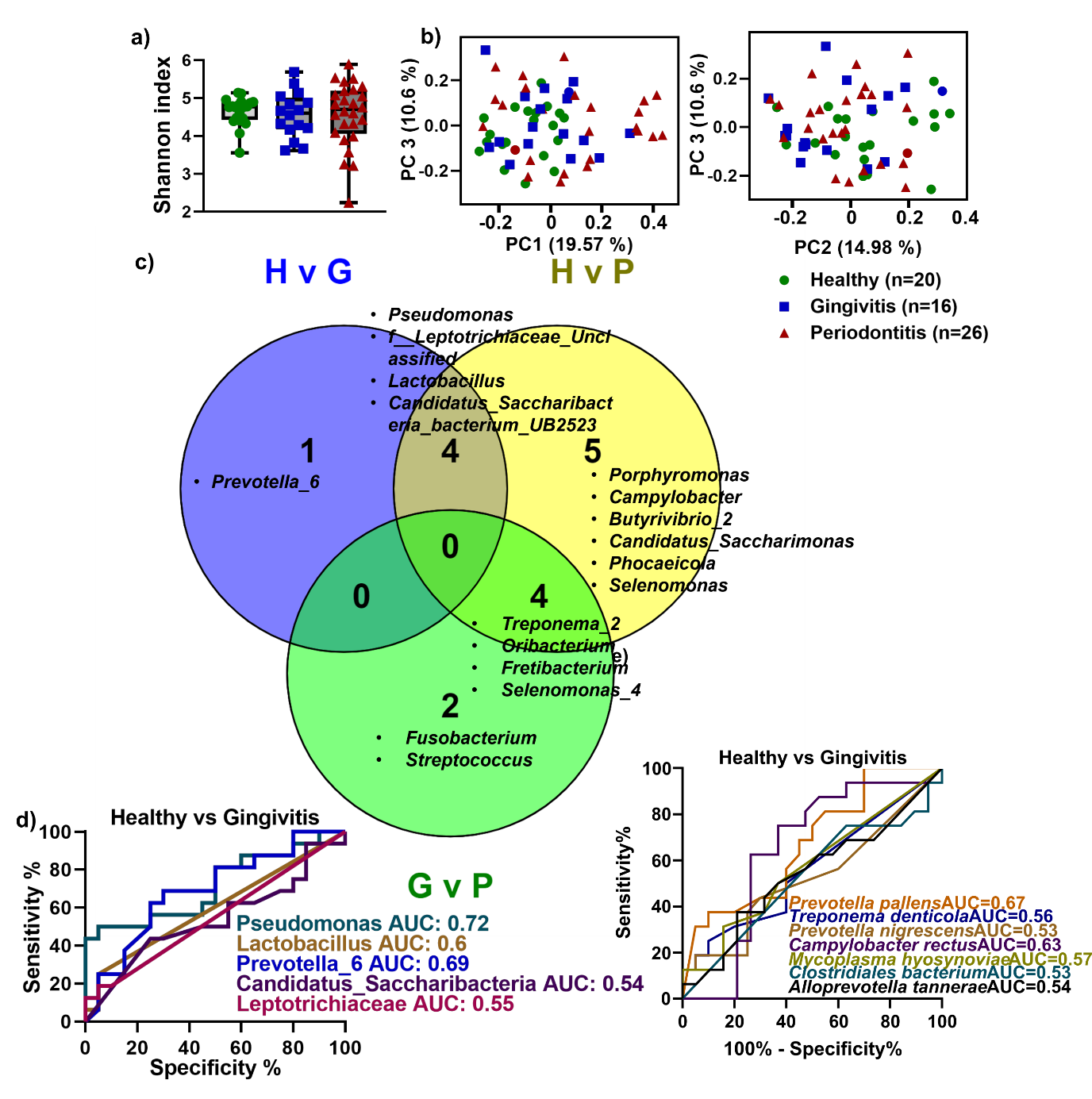
**

**Supplementary Figure 5.** a) Shannon diversity index and b) PCOA analysis for PC1/PC3 and PC2/PC3 (right). c) Common significant genus after comparison between groups using t-test. d) ROC curves for bacteria between healthy and gingivitis for genus (left)and species (right).

**
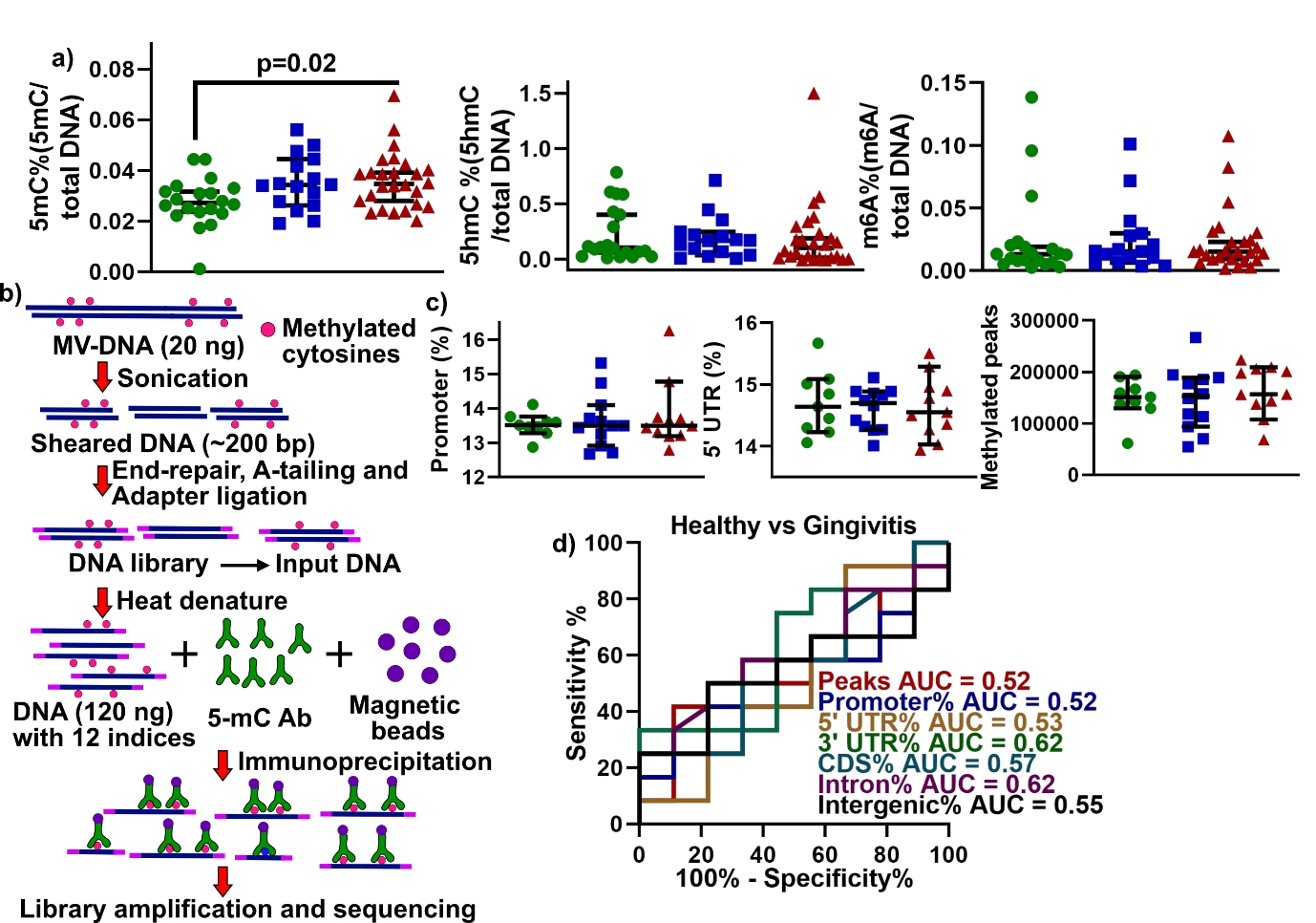
**

**Supplementary Figure 6. Global DNA methylation and commonly methylated genes after MeDIP-seq data analysis. a)** Global DNA methylation of 5mC, 5hmC and m6dA in 20 healthy, 16 gingivitis and 26 periodontitis. **b)** Schematic representation of the methylated DNA immunoprecipitation next-generation sequencing (MeDIP-seq) by using only 20ng of MV-DNA from each donor. **c)** No significant difference in promoter, 5’ UTR and methylated peaks between groups. **d)** The diagnosis power of global epigenome between healthy and gingivitis after AUC analysis.

**
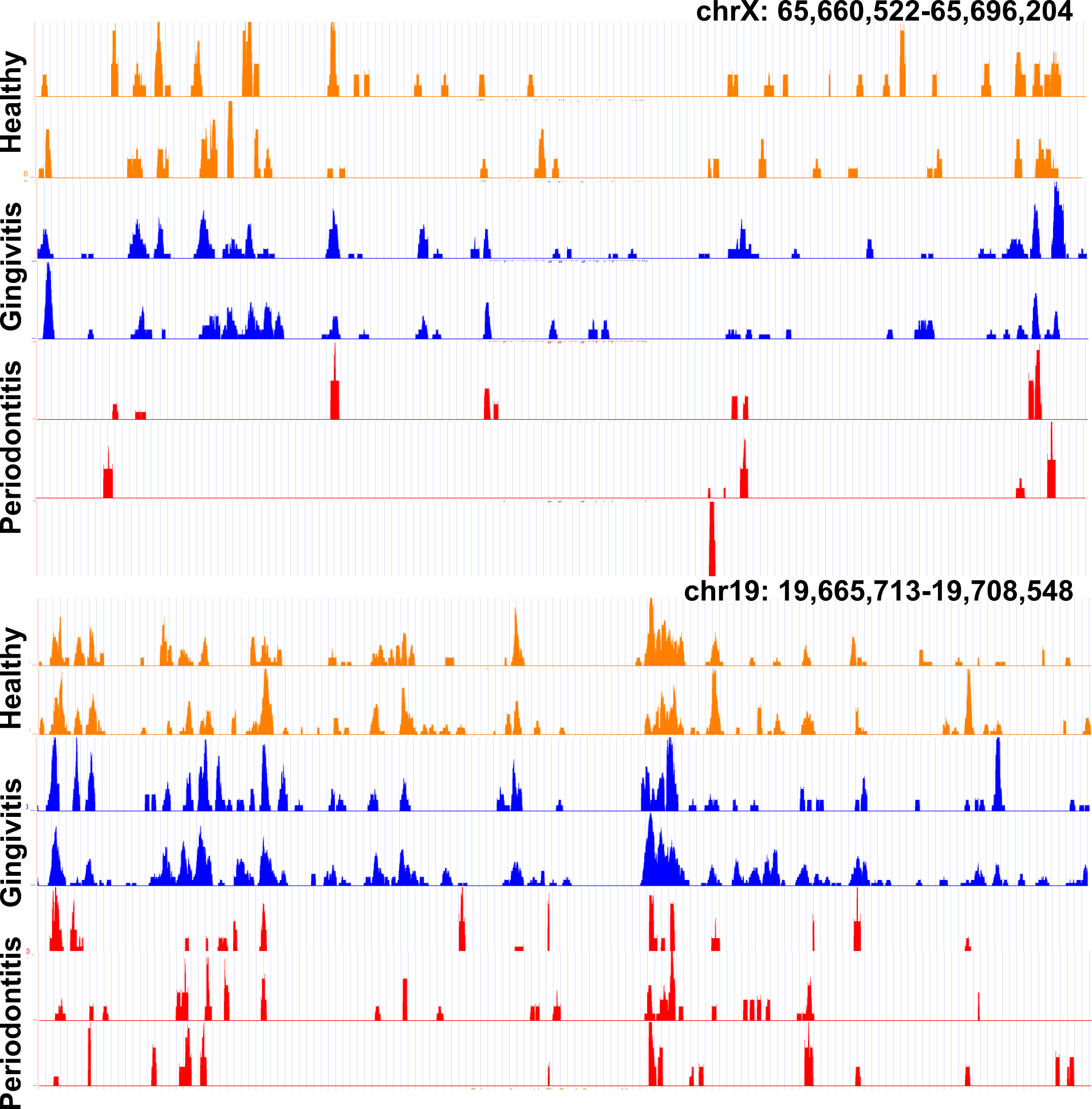
**

**Supplementary Figure 7. Browser representation of methylation patterns in chromosome X and chromosome 19.**

**
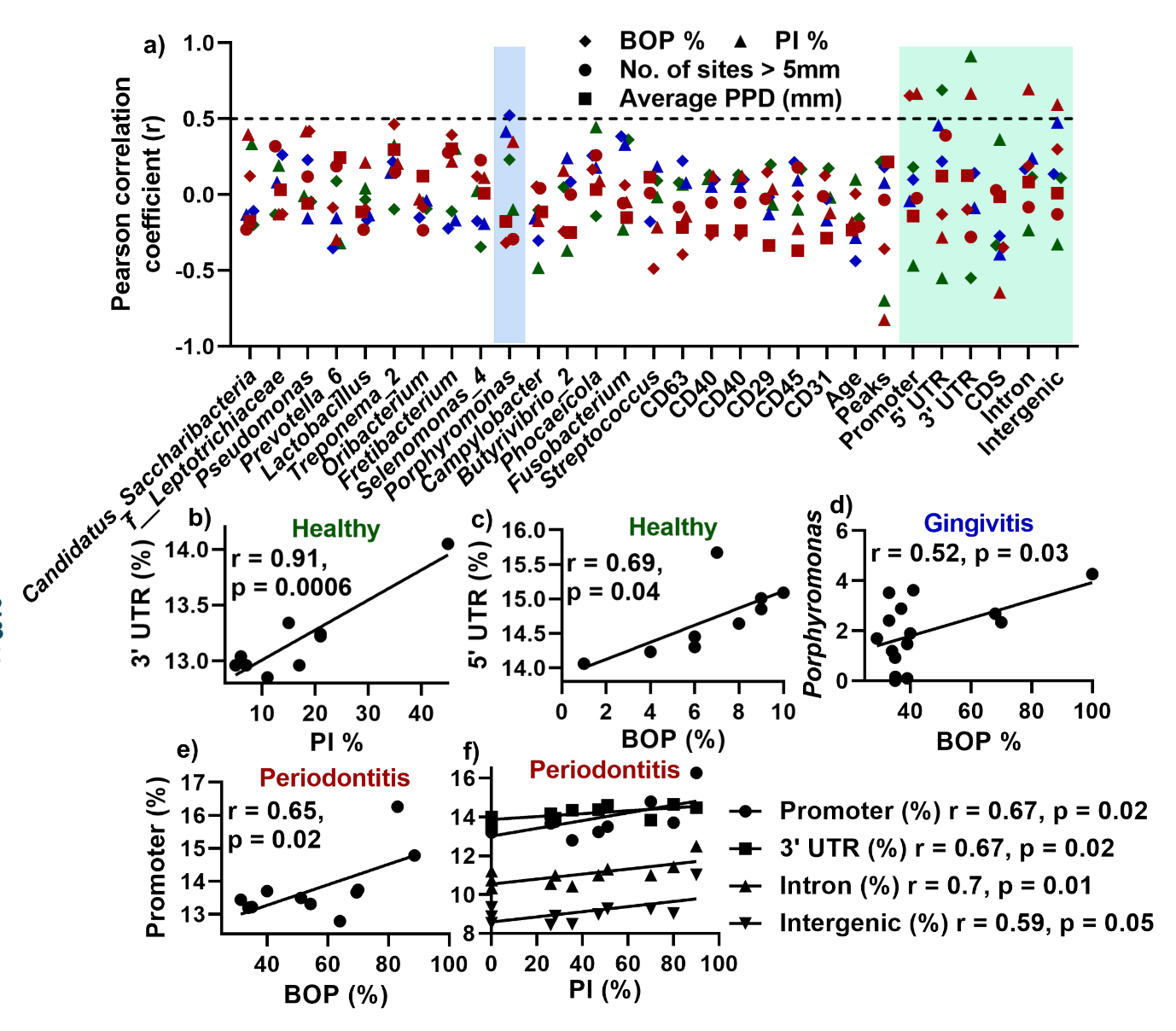
**

**Supplementary Figure 8. Pearson correlation between bacteria genus, CDs, age, methylation peak, global epigenome and clinical parameters. a)** MVs-methyl landscape and bacteria are positively associated with clinical parameters, PI% and BOP%, with significant association coefficients being higher than 0.5. Green dots-healthy, blue dots-gingivitis, red dots-periodontitis. **b-f)** Example of 3’TUR-PI% (b), 5’UTR-BOP% (c), *Porphyromonas*-BOP (d), promoter-BOP (e) and PI-methyl (f) in healthy, gingivitis and periodontitis patients.

**
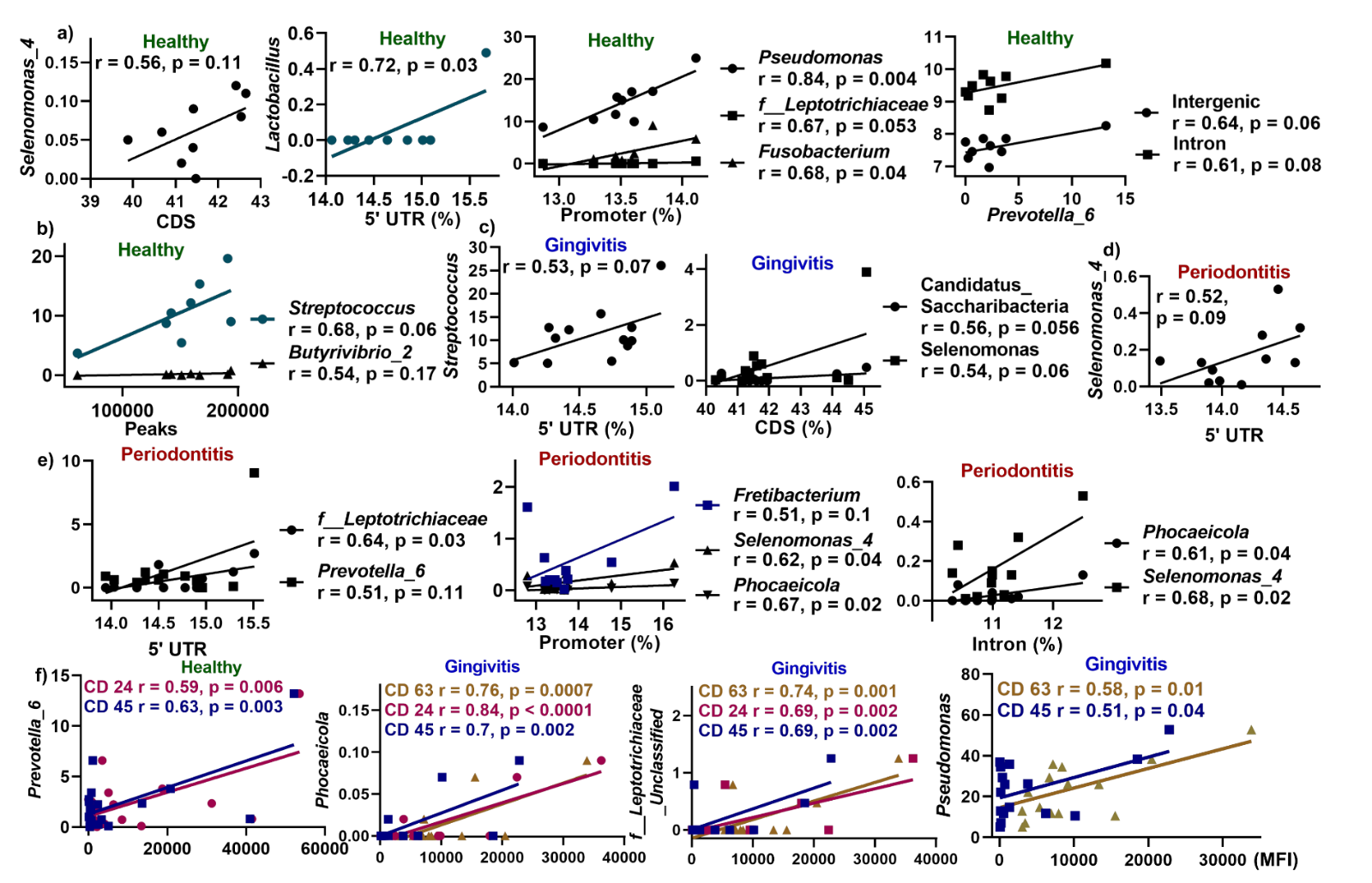
**

**Supplementary Figure 9. Person correlation between microbial and CDs (a, b) and global methylation landscape (c-e) in healthy, gingivitis and periodontitis patients.**

**
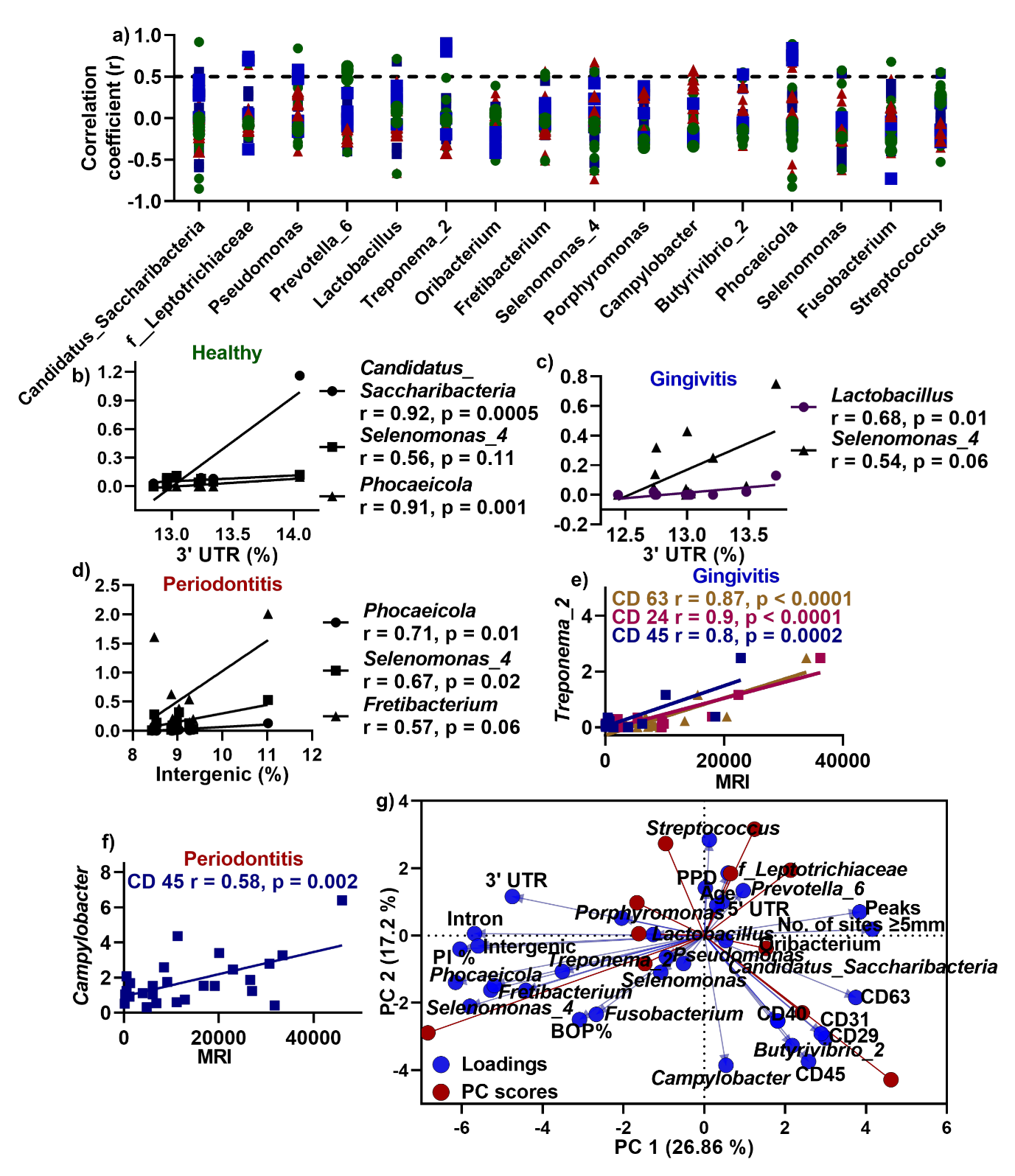
**

**Supplementary Figure 10. Correlation between different omics and clinical parameters that differentiate disease and healthy groups. a**) MV microbial associations with CDs-EVs and the global epigenome are different in gingivitis and periodontitis from periodontally healthy patients. Green, blue and red dots denote the correlation coefficient r values for healthy gingivitis and periodontitis participants. Dot line indicates r values > 0.5. **b-d**) Examples of microbial-methylation correlation that is significant in healthy (b), gingivitis (c) and periodontitis (d). **e-f**) Person correlation of microbial-CDs in gingivitis (e) and periodontitis (f). **g**) Biplot of the Principal Component Analysis (PCA) showing the data projected for the PC1 and PC2 planes. Correlated components and variables are located in the same quadrant. Close nearby points in the biplot represent samples with similar patterns. Connecting lines show the variables to each principal component based on loading and PC scores.

**References**

1. Han, P., et al., *Salivary Small Extracellular Vesicles Associated miRNAs in Periodontal Status-A Pilot Study.* Int J Mol Sci, 2020. **21**(8).

2. Han, P., et al., *Salivary Outer Membrane Vesicles and DNA Methylation of Small Extracellular Vesicles as Biomarkers for Periodontal Status: A Pilot Study.* Int J Mol Sci, 2021. **22**(5).

3. Tonetti, M.S., H. Greenwell, and K.S. Kornman, *Staging and grading of periodontitis: Framework and proposal of a new classification and case definition.* J Clin Periodontol, 2018. **45 Suppl 20**: p. S149-s161.

4. Han, P., et al., *Detection of Salivary Small Extracellular Vesicles Associated Inflammatory Cytokines Gene Methylation in Gingivitis.* Int J Mol Sci, 2020. **21**(15).

5. Théry, C., et al., *Minimal information for studies of extracellular vesicles 2018 (MISEV2018): a position statement of the International Society for Extracellular Vesicles and update of the MISEV2014 guidelines.* J Extracell Vesicles, 2018. **7**(1): p. 1535750.

6. Han, P., et al., *TNF-α and OSX mRNA of Salivary Small Extracellular Vesicles in Periodontitis: A Pilot Study.* Tissue Eng Part C Methods, 2023. **29**(7): p. 298-306.

7. Paolini, L., et al., *Fourier-transform Infrared (FT-IR) spectroscopy fingerprints subpopulations of extracellular vesicles of different sizes and cellular origin.* J Extracell Vesicles, 2020. **9**(1): p. 1741174.

8. Han, P., et al., *Effects of periodontal cells-derived extracellular vesicles on mesenchymal stromal cell function.* J Periodontal Res, 2023.

9. Koliha, N., et al., *A novel multiplex bead-based platform highlights the diversity of extracellular vesicles.* J Extracell Vesicles, 2016. **5**: p. 29975.

10. Szklarczyk, D., et al., *STRING v11: protein-protein association networks with increased coverage, supporting functional discovery in genome-wide experimental datasets.* Nucleic Acids Res, 2019. **47**(D1): p. D607-D613.

11. Li, H. and R. Durbin, *Fast and accurate short read alignment with Burrows-Wheeler transform.* Bioinformatics, 2009. **25**(14): p. 1754-60.

12. Robinson, M.D., D.J. McCarthy, and G.K. Smyth, *edgeR: a Bioconductor package for differential expression analysis of digital gene expression data.* Bioinformatics, 2009. **26**(1): p. 139-140.

13. Huang, D.W., B.T. Sherman, and R.A. Lempicki, *Systematic and integrative analysis of large gene lists using DAVID bioinformatics resources.* Nature Protocols, 2009. **4**(1): p. 44-57.
